# A One-Component Patchy-Particle Icosahedral Quasicrystal

**DOI:** 10.1021/acsnano.4c14885

**Published:** 2025-04-01

**Authors:** Eva G. Noya, Jonathan P. K. Doye

**Affiliations:** † Instituto de Química Física Blas Cabrera, Consejo Superior de Investigaciones Científicas, CSIC, Calle Serrano 119, 28006 Madrid, Spain; ‡ Physical and Theoretical Chemistry Laboratory, Department of Chemistry, University of Oxford, South Parks Road, Oxford OX1 3QZ, U.K.

**Keywords:** icosahedral quasicrystal, self-assembly, patchy particle, nanoparticles, computer simulations

## Abstract

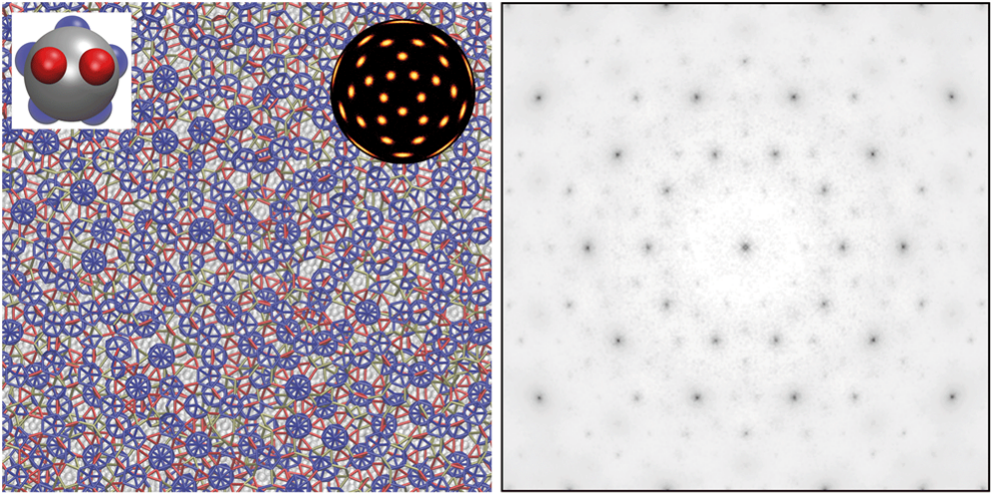

Designing particles that are able to form icosahedral quasicrystals (IQCs) and that are as simple as possible is not only of fundamental interest but is also important to the potential realization of IQCs in materials other than metallic alloys. Here we introduce one-component patchy-particle systems that in simulations are able to form face-centered IQCs that are made up of interconnected icosahedra. The directional bonding of the particles facilitates the formation of a network of bonds with icosahedral orientational order and hence quasiperiodic positional order. The assembled quasicrystals have similar energies to periodic approximants but are entropically stabilized by phason disorder. Their long-range quasiperiodic order is confirmed by a higher-dimensional analysis. These materials, which are predicted to have an almost spherical photonic band gap, can potentially be realized via protein design and DNA origami particles.

## Introduction

Quasicrystals (QCs) are structures with long-range structural order (as evidenced by sharp peaks in a diffraction pattern) but with no periodically repeating unit. This lack of translational symmetry allows quasicrystals to exhibit symmetries not possible in periodic crystals. For example, the first quasicrystal discovered in a material, namely an alloy of aluminum and manganese, had icosahedral symmetry.^1^ Subsequently, many other examples of icosahedral quasicrystals were discovered in metallic alloys,^2,3^ but intriguingly never in any other type of material.

Whether icosahedral quasicrystals can be realized in other materials is thus an open question. One way to begin to address this question is to use simulations and theory to better understand the requirements for particles to assemble into quasicrystals. One feature of quasicrystals is that indexing their diffraction patterns requires (at least) two integers per quasicrystalline dimension and in icosahedral quasicrystals the two inverse length scales are related by the golden ratio τ = (1 + √5)/2. Thus, one successful approach to design model particles that are capable of assembling into IQCs is to use isotropic potentials with complex radial forms that have features at multiple length scales.^4−9^ However, how to realize particles with these complex potentials is less clear.

An alternative design strategy is to instead use directional interactions that favor the formation of a phase with the desired global symmetry. This approach was first used to produce “patchy” particles that can assemble into dodecagonal quasicrystals^10−12^ and more recently was extended to produce patchy-particle IQCs.^13^ These original designs often required torsional terms, but new design strategies have recently been proposed to avoid this requirement, which may be hard to realize in experimental systems, but at the cost of introducing further particles types.^14^ One potential advantage of patchy particles over isotropic models is that the methods developed to design DNA origami particles^15−17^ or proteins^18^ that can form a variety of crystals through their directional interactions might be extendable to realize DNA or protein quasicrystals. Finding model IQC-forming systems that are as simple as possible, as well as being of fundamental interest, is also likely to aid their experimental realization. In ref (13) two types of patchy-particle IQC-forming system were developed, both of which were shown to be simplifiable to binary systems. Here, we go one step further by introducing a third patchy-particle system that can form an IQC. Remarkably only one particle type is required.

## Results

### Particle Design

Our basic design approach is to choose the patch geometry of the particles so that it matches the directions of the bonds in the different local environments in our target structure. For crystals this is relatively simple,^19^ however, there are two additional complexities for QCs: first, how to generate the target QC; second, as the number of different local environments in the IQC can be large, how to avoid the use of an unreasonably large number of particle types.

To generate an ideal target IQC we use the cut-and-project method.^20^ This involves the projection of a subset of the lattice points in a 6-dimensional hypercubic lattice onto an appropriately chosen 3-dimensional hyperplane. This hyperplane is termed the “parallel” space, and the 3-dimensional space orthogonal to this hyperplane is termed the “perpendicular” space. In particular, lattice points are projected if a 3-dimensional volume in perpendicular space (termed the “occupation domain”) centered on that point intersects the hyperplane. As parallel space represents an irrational cut through the hyperspace, the resulting structure cannot be periodic. This approach can be modified to generate a periodic crystal by instead choosing a hyperplane that has a rational slope. These periodic crystals are termed rational approximants. Previously, we have used body-centered and primitive hypercubic lattices.^13^ Here, we instead use a face-centered lattice.

**Figure 1:**
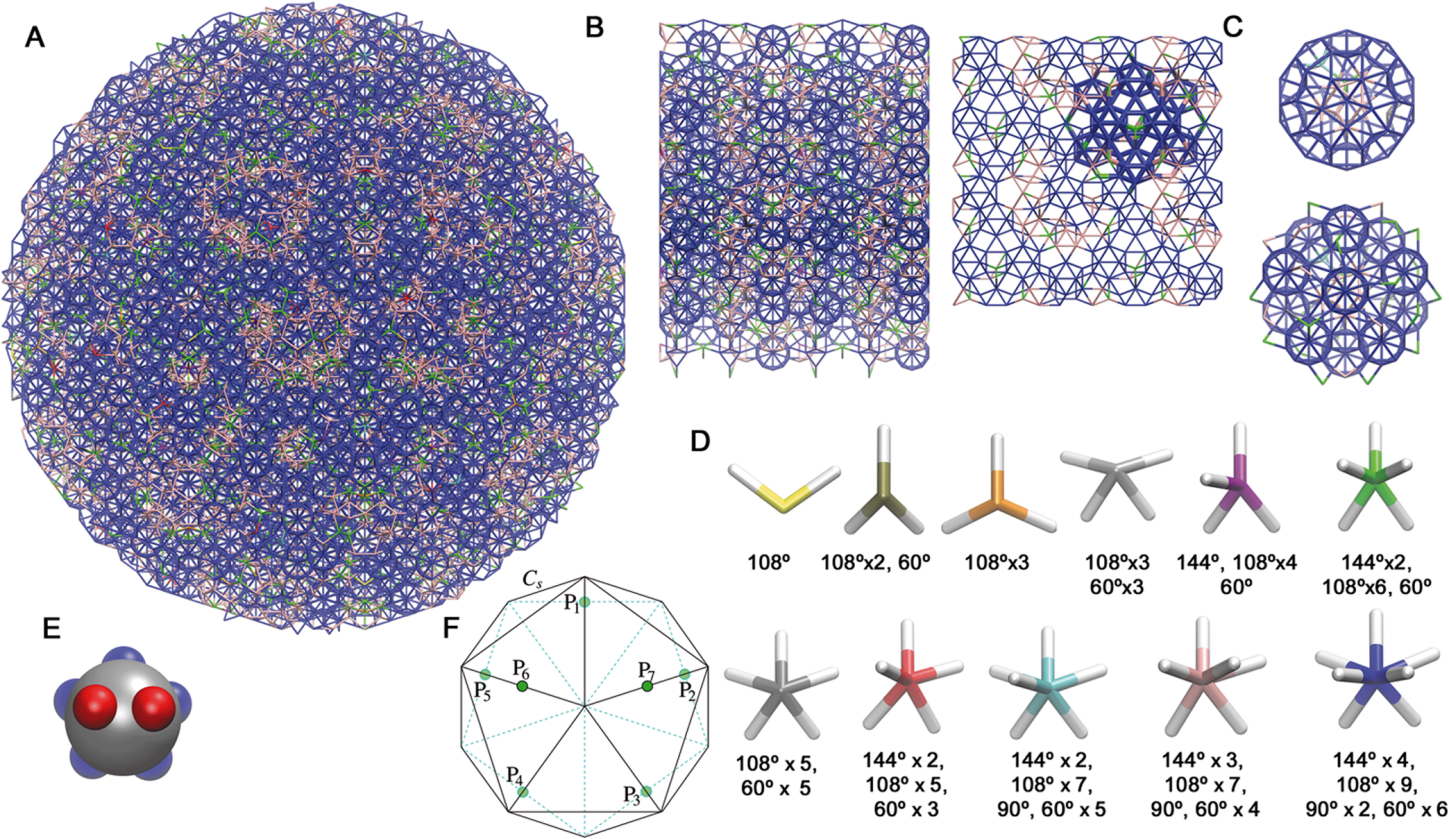
From ideal quasicrystal to patchy-particle design. (A) An ideal face-centered icosahedral (FCI) quasicrystal viewed along a 5-fold axis. (B) A 3/2 rational approximant viewed along a pseudo-5-fold axis and a 2-fold axis. (C) Example icosahedral clusters in the ideal quasicrystal: a rhombicosidodecahedron (top) and an icosahedra of icosahedra (bottom). These clusters are also present in the 3/2 approximant. The latter is also highlighted in B with thicker bonds. (D) Local environments in the ideal quasicrystal (with two or more neighbors). The colors match those used in A and B. All environments can be considered as subsets of the 7-coordinate environment. (E) The patchy-particle design that matches the 7-coordinate environment. (F) The relationship between the symmetry of the patchy particle and the *I*_h_ point group. The particle can be oriented so that its patch vectors **P**_**i**_ point along seven of the 2-fold rotational axes of *I*_h_. Edges on the back faces of the icosahedra are dashed and cyan. Similarly, for patch vectors on the back faces, the color shade is lighter and ringed in cyan rather than black.

An example ideal IQC produced by this approach is shown in Figure 1A. Also depicted is the 3/2 rational approximant to the IQC (Figure 1B). This approximant has 288 atoms per unit cell (285 disregarding zero-coordinated particles) and belongs to the *R*3 (146) space group. A common local motif in both structures is the simple 12-particle icosahedron. In some places in the ideal IQC and the 3/2 approximant, these icosahedra are even arranged into an icosahedron of icosahedra (Figure 1C); however, more often the requirements of long-range order lead to clusters involving some defective or incomplete icosahedra. Consequently, the number of different environments in the ideal IQC is large. The 11 environments with a coordination number of 2 or more are illustrated in Figure 1D; the environment with a coordination number of 7 is most common (69%) and 97% of the environments have a coordination number that is greater than or equal to 5. In the previous examples, we grouped together the environments that were subsets of each other and introduced particles corresponding to the highest coordination environment in each class,^13^ the hope being that, even though not all the patches would be used in the IQC, it would still represent the preferred structure for the patchy-particle system.

Here, all 11 environments form one class all being subsets of the 7-coordinate environment. Therefore, we explored the self-assembly behavior of one-component systems of the 7-patch particle depicted in Figure 1E (designated the 7P FCI model). Figure 1F illustrates the relationship of the patch geometry to the icosahedral point group *I*_h_. The particle can be oriented so that all 7-patches point along *C*_2_ axes of the group. The patches form two sets. The first set A consists of 5 patches related by one of the *C*_5_ axes of *I*_h_. These patches are typically involved in forming the intraicosahedral bonds. In fact, particles just having these five patches have been extensively explored as models of particles able to form finite complexes with high symmetry; they are able to assemble from a monomeric gas into a gas of icosahedral clusters.^21−23^ The second set B consists of two patches related by a mirror plane of *I*_h_ and are mainly responsible for intericosahedral bonds; these form connections between the edges of the icosahedra.

The interactions between particles are described by the same patchy-particle model as in refs^13,19^. In this pair potential, the interaction is described by a Lennard–Jones repulsive core and an attractive tail modulated by angle and possibly torsion dependent functions. The angular modulation term has a Gaussian form and is a measure of how directly two patches point at each other. The standard deviation of the Gaussian σ_ang_ is a measure of the angular width of the patch. The torsional modulation term describes the variation in the potential as either of the particles is rotated about the interparticle vector. It also has a Gaussian form that is centered at the preferred torsional angle. The parameter σ_tor_ is a measure of the torsional specificity of the interaction. To capture the symmetry of an environment, more than one preferred torsional angle can be defined. Unless otherwise stated we use σ_ang_ = 0.3 radians, as this provides a reasonable balance between the patches being sufficiently narrow to favor the target structure while not being so narrow that the kinetics of assembly is significantly hindered. When using torsionally specific interactions, we use σ_tor_ = 2σ_ang_. However, we found that adding a torsional term was not necessary to achieve IQC assembly in the current systems. All patches interact with each other, but we use the relative strength of the interactions between the two types of patches ϵ_BB_/ϵ_AA_ to help control the assembly. We set ϵ_AA_ = 1 and consider different values for ϵ_BB_. We always use ϵ_AB_ = (ϵ_AA_ + ϵ_BB_)/2. We use σ_LJ_ (the distance at which the Lennard–Jones potential is zero) as our unit of length, and the Lennard–Jones well depth ϵ_LJ_ as our unit of energy. Temperatures are given in reduced form, *T* * = *k*_B_*T*/ϵ_LJ_. The properties of the patchy interactions for the different models are fully tabulated in the Supporting Information (SI), as well as the precise mathematical form of the potential for the ring patches.

### IQC Self-Assembly

To explore whether our patchy-particle design could assemble into an IQC we used constant temperature Monte Carlo simulations starting from a low-density fluid. The temperature was chosen so that there would typically be nucleation and growth of a single cluster of the condensed phase. A key variable in the self-assembly behavior is ϵ_BB_/ϵ_AA_ as this affects the relative favorability of intra- versus intericosahedral bonds.

**Figure 2:**
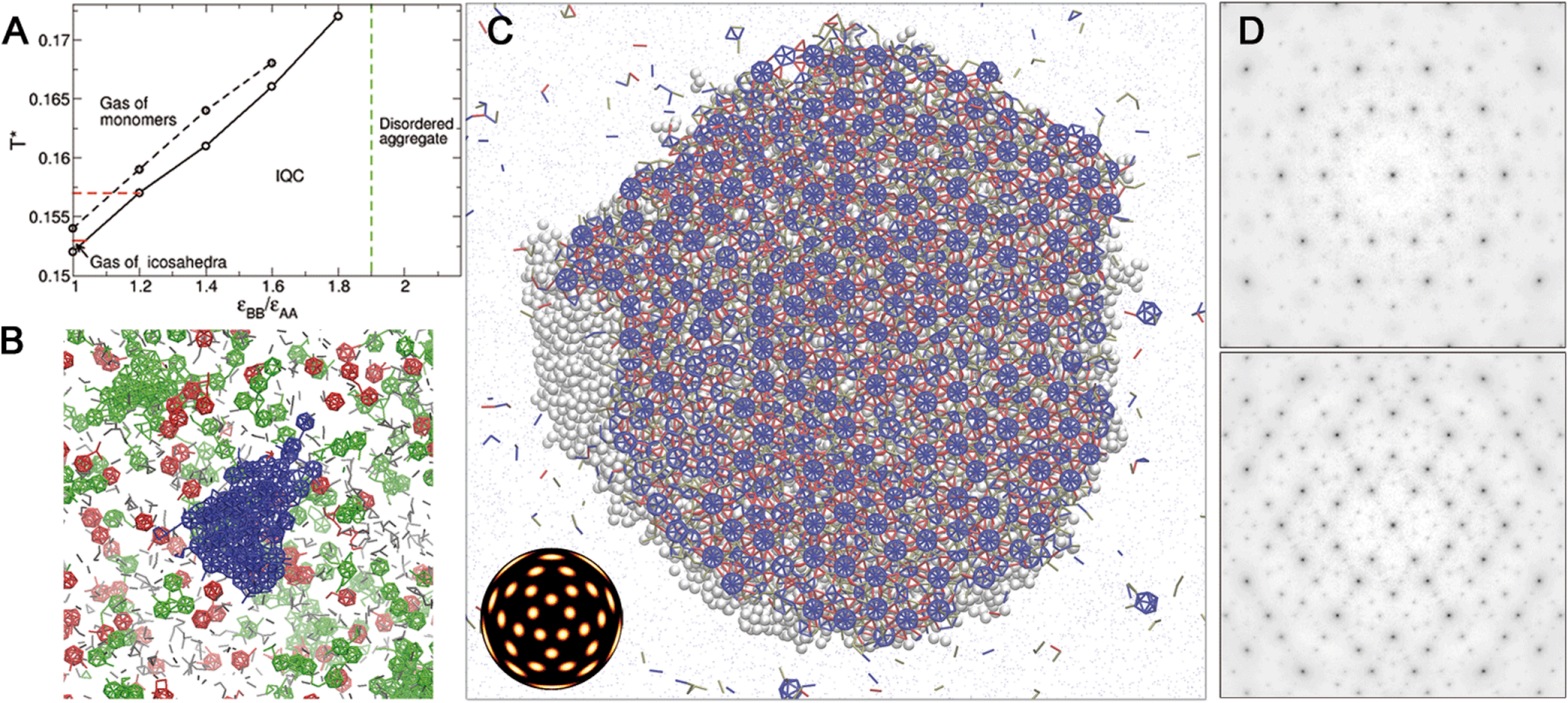
Assembly of the 7P FCI model. (A) Schematic assembly diagram as a function of ϵ_BB_/ϵ_AA_. Solid circles mark the highest temperature at which spontaneous assembly was observed for each ϵ_BB_/ϵ_AA_ on the time scale of our simulations, and open circles the temperatures at which the properties of the assembled IQCs were studied. The solid and dashed black lines are guides to the eyes. At ϵ_BB_/ϵ_AA_ = 1.0 the formation of the IQC was hindered by the assembly of multiple small aggregates. In this case we set the crystallization temperature as that for which the largest cluster was observed (that contained about 800 particles, shown in (B). For ϵ_BB_/ϵ_AA_ > 1.9 particles assembled into a disordered aggregate. The solid red horizontal line demarcates the center of the transition from a gas of monomers to a gas of icosahedra that is estimated from simulations of particles with five patches of type A (see Supporting Information and Figure S9 for further details). The dashed red line provides an indication of the width of this transition by marking the temperature at which 30% of particles are in icosahedra. (B) View of a typical configuration at ϵ_BB_/ϵ_AA_ = 1.0 and T* = 0.152. The largest cluster is shown with blue bonds, clusters containing more than 15 particles with green bonds, those with between 9 and 15 particles with red bonds and those with less than 9 particles with gray bonds. (C) Cut through an assembled 45 000-particle cluster obtained at ϵ_BB_/ϵ_AA_ = 1.2 and T* = 0.159. Bonds close to the cut plane are colored using the scheme: AA bonds in blue, BB bonds in red, and AB in tan. Other particles in the cluster are shown as gray spheres and particles in solution as gray dots. The BOOD shows that all the bonds are directed along the 2-fold axes of *I*_h_. (D) Diffraction pattern projected along the 5-fold and 2-fold symmetry axes.

Figure 2C shows a cut through a 45 000 particle cluster grown at ϵ_BB_/ϵ_AA_ = 1.2. The diagram is colored to highlight the icosahedra that are held together by AA bonds. It can be seen that the icosahedra are all oriented with their local 5-fold axes of symmetry out of the plane. The global orientational order is confirmed by the bond-orientational order diagram (BOOD). The BOOD shows that the bonds are oriented along the 2-fold directions of *I*_h_, as expected from the patchy-particle geometry (Figure 1F). The quasicrystallinity of the cluster is confirmed by the diffraction patterns (Figure 2D); they both show that the cluster has long-range order and that it possesses 5-fold symmetry around the relevant axes. A comparison of the diffraction pattern to that for the ideal quasicrystal (Figure S3) confirms that they share the same set of major peaks, and an indexing of the pattern (Figure S4) confirms that the quasicrystal is face-centered icosahedral. Thus, we have discovered a one-component patchy-particle IQC-forming system. It is noteworthy that this was achieved without introducing a torsional component to the potential to disfavor possible competing crystals; controlling just the geometry of the local coordination shell through the patch geometry is sufficient to ensure IQC formation. The lack of competing simple crystals may partly reflect just the number of patches; it has previously been noted that there is a paucity of crystals with an average coordination number close to 7.^8^

We obtained the most ordered IQC clusters at ϵ_BB_/ϵ_AA_ = 1.2. At ϵ_BB_/ϵ_AA_ = 1.0, however, IQC self-assembly was not observed. Under these conditions, instead of forming an IQC direct from the low-density fluid, the particles first start to assemble into isolated icosahedra. Although some of these icosahedra further assemble into smallish aggregates, the majority remain as icosahedra and the formation of an IQC is very difficult; Figure 2B shows an example of the configurations that result. The problem is that when the icosahedra form the orientation of the B patches on the surface will effectively be random and unlikely to match that needed for growth of the quasicrystalline phase. Although the system is not completely dynamically arrested — e.g., there is a dynamic equilibrium between monomers and icosahedra — the reorientational dynamics of the assembled particles is too slow for significant IQC formation on the simulation time scales. Note that this lack of assembly is purely due to kinetic factors; if the IQC grown at ϵ_BB_/ϵ_AA_ = 1.2 is then simulated at ϵ_BB_/ϵ_AA_ = 1.0 it is stable under the same conditions as in Figure 2B.

A schematic assembly diagram as a function of ϵ_BB_/ϵ_AA_ and temperature is shown in Figure 2A. It shows the regions of stability of the monomeric fluid, the fluid of icosahedra and the icosahedral quasicrystal, with the latter being increasingly stabilized with respect to the fluid phases as ϵ_BB_/ϵ_AA_ increases. Only beyond about ϵ_BB_/ϵ_AA_ = 1.3 is there a direct transition between the monomeric fluid and the quasicrystal. At ϵ_BB_/ϵ_AA_ = 1.2 there is still a competition between the formation of isolated icosahedra and the IQC, but unlike at ϵ_BB_/ϵ_AA_ = 1.0 it just slows down assembly rather than prevents it (Figure S8B); indeed, the slower growth might be one of the reasons that the IQC at ϵ_BB_/ϵ_AA_ = 1.2 is relatively so ordered with its properties most closely matching that of a simulated ideal IQC (Table S4). IQC formation is observed up to ϵ_BB_/ϵ_AA_ = 1.8, but beyond this disordered aggregates lacking icosahedra instead form because of the comparative weakness of intraicosahedral bonding.

**Figure 3:**
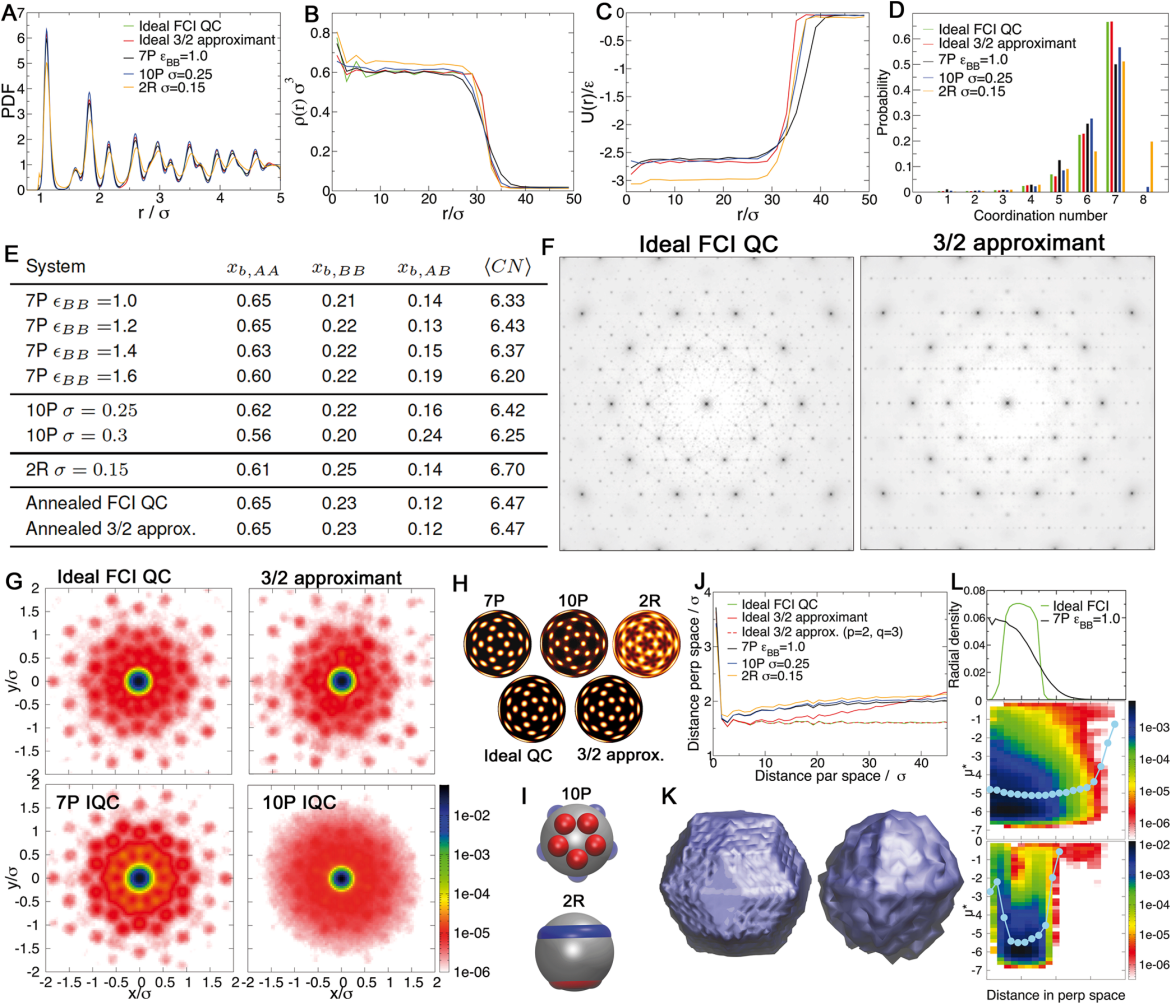
Comparison of the assembled quasicrystals (7P IQC, 10P IQC and 2R IQC) and the ideal FCI QC and a 3/2 approximant simulated with the 7P model. In these five simulations, ϵ_BB_/ϵ_AA_ = 1 (the starting point for the FCI 7P annealing simulation was a cluster grown at ϵ_BB_/ϵ_AA_ = 1.2). (A) Pair distribution functions. (B) Density ρ(*r*) and (C) energy *U*(*r*) as a function of the distance from the center of the cluster *r*. (D) Coordination number distributions. (E) The average coordination number (⟨*CN*⟩) and the fraction of AA, BB and AB bonds (*x*_AA_, *x*_BB_ and *x*_AB_). For the 7P model, results for interactions strengths ϵ_BB_/ϵ_AA_ = 1.0, 1.2, 1.4, and 1.6 (all with σ_ang_ = 0.30) are also given. For the 10P model data is shown for σ_ang_ = 0.25 and for σ_ang_ = 0.30 with a torsional term. (F) Diffraction patterns of the annealed ideal FCI QC and the 3/2 rational approximant projected along the 5-fold axis. (G) Van Hove correlation functions. (H) BOODs for all 5 systems. (I) The 10P and 2R patchy particles. (J) Phason strain. For the approximant the distance in perpendicular space has zero slope if the lifting is done taking *p* = 2 and *q* = 3 in perp space. (K) Shape of the occupation domain in the annealed ideal FCI QC (left) and the assembled 7P IQC (right). (L) Radial density in perpendicular space (top panel). Probability density of the occupation domain as a function of distance in perpendicular space and particle’s energy (u*) for (middle panel) the 7P IQC (ϵ_BB_/ϵ_AA_ = 1.0) and for (bottom panel) the annealed ideal FCI QC. Blue line and symbols indicate the average energy as a function of distance in perp space.

In our search for the simplest IQC-forming patchy-particle system it would have been preferable if all the patch–patch interactions could be of equal strength. We therefore explored possible ways that the kinetic traps that prevent assembly at ϵ_BB_/ϵ_AA_ = 1 might be overcome. Our solution was to instead use a particle with five equivalent B patches (Figure 3I), named 10P FCI model. Such particles still effectively have a maximum coordination number of seven as it is not possible for two adjacent B patches to be simultaneously involved in strong bonds since the angle between the patches is 36° and the neighboring particles would be significantly displaced from the minimum energy positions to avoid overlaps (Figure S10). However, now once an icosahedron forms the directions in which BB bonds can form are no longer predetermined, as any two of the five B patches can be used. Our initial simulations with such particles led to the formation of a liquid droplet; note that it is well-established that increasing the number and width of patches increases the temperature of the liquid–vapor critical point.^24^ We therefore made the patches narrower setting σ_ang_ = 0.25 radians. This led to the successful assembly of an IQC (Figure S7). Although the addition of the extra 3 B patches was motivated by their potential effect on the dynamics, they also affect the thermodynamics and assembly now actually occurs direct from the monomeric vapor (Figure S8). This is because the increase in the number of B patches entropically stabilizes the IQC (there are more possible particle orientations that are compatible with the IQC) but has no effect on the transition to a gas of icosahedra. We also note that adding torsional interactions rather than making the patches narrower also sufficiently disfavors the formation of disordered aggregates that an IQC grows direct from a low-density fluid (Figure S8).

Additionally, we tested the assembly of a third design in which each of the two sets of 5 patches in the 10P model was replaced by a ring patch (Figure 3I). We designate this model 2R FCI. The width of the ring patches was reduced to σ_ang_ = 0.15 radians to avoid stabilization of the liquid phase. This less specific design is still able to form an FCI QC (Figure S7), albeit somewhat less ordered than for the QCs assembled from the 7P and 10P models.

### Analysis of Annealed Samples

Besides analyzing the structural and dynamic properties of the 7P, 10P and 2R types of assembled IQCs, we also constructed patchy-particle versions of the ideal IQC and the 3/2 rational approximant and compared the properties of the five systems averaged over long annealing simulations (Figure 3). The initial configurations were built by placing particles at the lattice sites of the ideal configurations of the IQC and the 3/2 rational approximant. Particle orientations were obtained by using a Monte Carlo code that, for each particle, searches for the orientation that minimizes the deviation of the particle’s patches from the first coordination shell in the ideal configuration. These configurations are then linearly heated from a reduced temperature of 0.01 to 0.154 over a 1 million MC cycles simulation. The ideal FCI QC and its rational approximant are then allowed to evolve for at least further 4 million MC cycles at T* = 0.154, using the 7P model with ϵ_BB_/ϵ_AA_ = 1. The structural properties of the assembled 7P, 10P, and the annealed ideal IQC and 3/2 approximant are very similar, including the radial distribution functions and the bulk densities and energies (Figure 3A–C). The latter is particularly noteworthy, as it means that the IQC is likely to be thermodynamically stable over a significant range of temperature due to its greater entropy. The absence of a significant energetic advantage for the approximant also helps to further explain why it is never observed in the assembly simulations.

Consistent with the similar energies the average coordination number for the ideal IQC and the approximant are effectively the same and only 8% less than the maximum possible value of 7 (again helping to explain why no alternative crystal forms were observed). Furthermore, the most ordered examples of the assembled 7P and 10P IQCs have a coordination number that is only a bit less and with a similar proportions of inter- and intraicosahedral bonding (Figure 3E). The 2R IQC differs from the other four systems in that the reduced angular constraints associated with the ring patches allow a significant fraction of particles to achieve a coordination number of 8, and hence a higher average coordination number, higher density and lower energy. The spots in the BOOD are also no longer isotropic, but have features associated with the angular vibrations about the particles’ symmetry axes being energetically less costly (Figure 3H).

The order in the approximant is very similar to that of IQCs. For example, the deviations in the BOOD from icosahedral symmetry are not apparent to the eye (in fact bonds are ∼5% more likely along the *C*_2_ axes along *x*, *y* and *z* (Figure S12)) and it is only in the weaker higher-order peaks in the diffraction pattern that the deviation from long-range 5-fold order is evident (Figure 3F).

The Van Hove correlation functions show that particle hops are possible in the IQCs and the approximant and exhibit (approximate) icosahedral symmetry. It is also noticeable that the structure in the Van Hove correlation function for the 10P and 2R IQCs is significantly less well-defined; the extra patches in 10P and the ring patches in 2R seem to allow greater freedom in the relative particle motion (Figure 3G). In the previous multicomponent patchy-particle IQCs,^13^ although the “matrix” particles exhibited similar mobility to the IQCs here, the particles associated with the (rhombic triacontahedral or dodecahedral) clusters tended to be relatively immobile, preventing significant structural change. The one-component nature of the current system is likely to allow easier structural relaxation; however, we should note that the particle mobility in the assembled quasicrystals decreases as a function of simulation time (Figure S13), presumably due to the annealing out of mobility-facilitating defects.

Another way to analyze the assembled IQCs is to do the reverse of the cut-and-project procedure that was used to generate our starting ideal IQC. In this “lifting” procedure every particle in the IQC is mapped onto a lattice point in 6D space. This can be achieved because the 6D interlattice vectors corresponding to each of the 30 bond directions (along the *C*_2_ axes of *I*_h_) in the ideal IQC are known. Thus, by iterating through the bond network all particles can be “lifted”. Important to this mapping being well-defined for the assembled IQCs is the absence of dislocations in these systems.

The lifted 6D coordinates occupy the face-centered lattice sites as expected. Indeed it follows that an IQC that exclusively has bonds along the 2-fold directions must be face-centered, as a vector in parallel space that is along a 2-fold direction can only be generated from a projected 6D lattice vector that has nonzero components along an even number of lattice directions.

This procedure allows the quasiperiodicity of our structures to be assessed. Figure 3J shows how the distance between the lifted lattice points in perpendicular space depends on their separation in parallel space. Quasicrystals are said to have zero phason strain if the slope of this plot is zero.^6,25^ This will trivially be the case for the ideal IQC generated by the cut-and-project method and still holds after this ideal IQC is annealed at finite temperature. By contrast, the 3/2 approximant has a clear linear phason strain, as expected from the different slope of the hyperplane used to generate this approximant. This phason strain is coherent in the sense that if the perpendicular distance was measured perpendicular to this rational hyperplane the slope would be then reduced to zero (the *p* = 2, *q* = 3 line in Figure 3J). For random tiling quasicrystals, the entropy is maximal for zero phason strain.^26^ But if a quasicrystal has quenched-in disorder (e.g., due to rapid solidification) this inability to reach equilibrium can lead to a finite phason strain.^27^ In our assembled IQCs the phason strain is approximately zero, further confirming their quasicrystallinity (Table S5).

The limiting values of the separation in perpendicular space are greater for the assembled IQCs than the ideal IQCs. This is as expected and reflects the greater phason disorder in the assembled systems (Table S5). The kinetically, and probably also thermodynamically, preferred state of the assemblies involves inherent disorder.

The occupation domain for the IQCs can be obtained by projecting the lifted lattice points into perpendicular space. For the ideal IQC the occupation domain retains its rhombic-triacontahedral shape on annealing (Figure 3K) but for the assembled IQCs the occupation domain is larger and more diffuse consistent with its greater phason disorder. In Figure 3L we show how the energy of the particles depends on their distance from their center of the occupation domain. Interestingly, for the assembled quasicrystals a particle’s energy is relatively independent of its position within the occupation domain. This also occurs in the assembled IQCs from our previous work^13^ (see Figure S16), but contrasts with the IQCs in ref (6). This feature helps to explain why the assembled FCI QCs have a similar energy to the approximant and the ideal IQC even though they have significant phason disorder. It also suggests that the driving force for their zero phason strain is likely to be entropic. This is somewhat similar to the random tiling model of quasicrystals.^26^ However, the configurations of the IQCs cannot be simply divided into a set of tiles with identical particle decorations; instead the contributors to the configurational entropy are more diverse.

## Conclusions

In summary, we have demonstrated that it is possible to assemble IQCs from one-component patchy colloidal systems. In some sense, our particle designs are somewhat akin to a patchy-particle “einstein” (an einstein is a monotile that can cover the plane aperiodically^28,29^). However, in our particle-based models, the assembled IQCs cannot be straightforwardly interpreted in terms of a few tiles with identical particle decorations that completely fill space.

In the current FCI examples, the predominant motif is the 12-particle icosahedron. Thus, the assembled IQCs share some similarities to icosahedral glass models^30,31^ which are obtained by irreversible aggregation of icosahedra with fixed orientation. However, in the patchy-particle IQCs, there are also incomplete icosahedra and a small fraction of particles that are not associated with icosahedra. Furthermore, as the growth is not irreversible, the particles, and hence the icosahedra, can seek to optimize their interactions. These additional features allow the system to exhibit approximately zero phason strain.

One important lesson from the icosahedral glass models was that global orientational order can lead to IQC-like order (albeit perhaps not with sharp Bragg diffraction peaks). Our particle-based models go further and show that directional interactions that locally favor a given global orientational order (in this case *I*_h_ symmetry), but without a second length scale, can be sufficient to generate long-range quasiperiodic order.

Although not actually proven here, given their competitive energetics and their additional entropy compared to a crystal, the assembled IQCs are likely to be thermodynamically stable over a wide temperature range. Random-tiling models^26^ are the archetypal models for entropically stabilized quasicrystals, where the entropy of the random tiling comes from the many different ways that the tiles can be combined with negligible energy cost. However, our particle-based IQCs cannot be simply mapped onto a small set of particle-decorated tiles due to the other types of disorder that are present, and these additional sources of entropy are likely to further stabilize the IQCs.

A pertinent question is whether these models can be translated into an experimentally realizable system. Despite advances in the synthesis of patchy colloids,^32^ producing particles with the required patch geometry and selectivity is probably beyond current capabilities. Both features can be more easily controlled using DNA nanotechnology, a field that has experienced huge advances in recent years.^33^ Specific interactions between DNA origami can be induced on the basis of shape complementarity^34^ or through the association of single-stranded “sticky ends”. This strategy has already been exploited to assemble finite clusters,^35^ one-dimensional assemblies^36^ and even three-dimensional crystals.^15−17^ We envision that one possible route to build our particle designs would be to use DNA origami icosahedra^16,37^ with single-strands to mediate edge-to-edge bonding between the relevant seven edges, thus mimicking the 7P particles. This would be quite similar to ref (16) where pyrochlore crystals were grown using DNA origami icosahedra but where the bonding was between selected vertices. Another possible route to obtain experimental analogues of our patchy-particle designs is to leverage the recent advances in computational protein design.^38^ For example, a general approach to program proteins to assemble into crystals with predefined symmetries has been recently developed.^18^ The higher symmetry of the 10P particles make them a more attractive target for protein design. Further details concerning potential experimental realization are given in Section S3 in the Supporting Information.

Being able to experimentally assemble an icosahedral quasicrystal from nanoparticles is an appealing goal in itself and also for practical applications. Given the high point group symmetry of IQCs, if assembled at the appropriate length scales, these structures can exhibit a small spherically symmetric optical band gap.^39^ Notably, it has been recently shown that the suitable length scales can be achieved for the assembly of a diamond lattice from DNA origami particles.^17^ In light of these recent experimental advances, the prospects of experimentally achieving a photonic icosahedral quasicrystal through directional bonds seem promising.

## Methods

### Ideal Quasicrystals

As the target structure, we used an ideal FCI QC generated with the cut-and-project method.^20^ Aperiodic structures can be produced by projecting a cut of a higher dimensional periodic lattice onto an hyperplane that crosses the lattice with an irrational slope. The higher dimensional space is divided into two orthogonal subspaces (the parallel and perpendicular spaces). Aperiodic structures can be produced by projecting the nodes of the higher dimensional lattice that fall within a region of perpendicular space (or occupation domain) onto the parallel space. To obtain an icosahedral quasicrystal we need to use a six-dimensional lattice, which is the lowest dimension in which it is possible to have a periodic lattice with icosahedral symmetry. Indeed, there are three lattices that fulfill this requirement: the primitive, the body-centered and the face-centered hypercubic lattices. In our previous work,^13^ we used as target structures IQC obtained from projection of the primitive and body-centered hypercubic lattices. Here, we generate an ideal IQC by projection of a face-centered hypercubic lattice using the canonical occupation domain (i.e., using the projection of the 6D unit cell onto perpendicular space as the occupation domain). Using the same convention as in ref (6), the basis matrix is given by
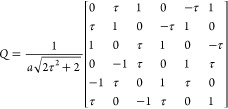
1
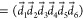
2The canonical occupation domain is a triacontahedron with a circumsphere of radius . Numerical errors that arise when the projections of some points lie exactly at the boundary conditions can be avoided using an algorithm based on integer numbers, as described in ref (40). Note that this problem can also be avoided by applying a shift to perpendicular space and leads to equivalent structures (i.e., belonging to the same local isomorphism class^20^). The canonical FCI QC tiling obtained using this procedure is depicted in Figure 1.

Besides the FCI QC we also generated some of the lower order rational approximants using the shear method.^20^ These periodic structures can be obtained by changing the irrational cut of the hyperspace to a rational one. This change of slope can also be seen as a shear transformation of the lattice in the higher dimensional space, defined by the 6 × 6 *A*^⊥^ matrix. The rational slopes are chosen as the quotient between two consecutive integer numbers of the Fibonacci series (*p*, *q*), which are then used to designate the order of the approximant. Thus, a lattice position of the 6D lattice, **r**, is transformed according to
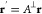
3Choosing the approximant basis vectors to coincide with the cubic axes pointing to *p*(*d⃗*_3_ + *d⃗*_6_) + *q*(*d⃗*_2_ – *d⃗*_5_), *p*(*d⃗*_2_ + *d⃗*_5_) + *q*(*d⃗*_1_ – *d⃗*_4_) and *p*(*d⃗*_1_ + *d⃗*_4_) + *q*(*d⃗*_3_ – *d⃗*_6_), and imposing the condition that their components in perpendicular space vanish, the shear matrix is given by
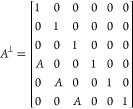
4with *A* = (*q* – τ *p*)/(*p* + τ *q*). The lattice parameters of the *p*,*q*-approximants are given by
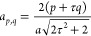
5It can be easily shown that this transformation is equivalent to replacing 1 by *p* and τ by *q* in the last three rows of the basis matrix (eq 1). Using this transformation we obtain a series of hexagonal approximants belonging to space group R3 (number 146 in the crystallographic tables). Among these, we have chosen to perform simulations for the 3/2 approximant. The structure considered was obtained by applying a shift of (0.4, 0.4, 0.4) in perpendicular space, as this leads to a slightly higher average coordination number. The unit cell of the 3/2 approximant generated contains 288 particles, 285 if zero-coordinated particles are disregarded.

### Simulations

The assembly behavior of the model systems was investigated by Monte Carlo simulations in the canonical ensemble. Simulations were performed with a bespoke GPU parallel Monte Carlo code, using a checker board algorithm.^41^ The assembly behavior was initially explored using a cubic simulation box with 20,000 particles at a density ρ = 0.10 σ_LJ_^3^. Simulations were carried out at a temperature at which nucleation occurred via a single solid seed in reasonable simulation times (typically less than 1–3 million MC cycles, where one MC cycle amounts to an average of one move attempt per particle). In these conditions, a roughly spherical solid cluster forms in coexistence with gas. In this way, the formation of defects associated with the incommensurability of the assembled quasicrystal with the cubic periodic box is avoided. Once a sufficiently big solid cluster was obtained (typically containing 15,000–18,000 particles), it was inserted in a larger simulation box containing a low density fluid so that the total number of particles is about 100,000 particles. Simulations in these larger systems are performed at a higher temperature to avoid the nucleation of additional solid clusters in the fluid. The system was then allowed to equilibrate until solid–vapor equilibrium is reached, after which another 6–8 million MC cycles were performed to take averages.

Besides the assembly simulations, we also studied the thermodynamic behavior of particle-based models of the ideal FCI QC and 3/2 approximant. The initial particle orientations in these configurations were obtained using a Monte Carlo code that for each particle searches for the particle’s orientation that provides the best alignment of its patch vectors with the interparticle vectors to its first coordination shell. Spherical clusters with about 80,000 particles and a radius of about 30 σ were inserted in a cubic simulation box of edge 100 σ surrounded by a gas of particles with a number density of about ρ* = 0.02 to mimic the equilibrium conditions found for the assembled FCI QCs. These configurations were progressively heated from T* = 0.01 up to T* = 0.154 during the course of a one million Monte Carlo cycles simulation, and then equilibrated for at least 4 million MC cycles. These systems were modeled with the 7P design and all patch–patch interactions having the same strength, ϵ_AA_ = ϵ_BB_ = ϵ_AB_ = 1.

### Analysis

Particles belonging to the solid clusters were identified using a cluster search algorithm^42^ with the convention that two particles are nearest neighbors if the energy between them is lower than −0.2 ϵ_LJ_. The structure of these solid clusters was characterized by evaluating the pair distribution function, the radial density and energy profiles, the coordination number distribution and the bond orientational order diagram (BOOD). The BOOD is a plot of the first coordination shell of each particle on a unit sphere, which is subsequently projected in the plane using an area-preserving Lambert projection. The single-particle dynamics was studied by evaluating the Van Hove autocorrelation function, measured after 1 million MC cycles:
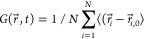
6where *r⃗*_*i*,0_ and *r⃗*_*i*_ are, respectively, the initial and current positions of particle *i*. To avoid surface effects, only the inner part of the cluster (i.e., particles within a distance of 25 σ_LJ_ of the cluster center of mass) is considered in the calculations.

The long-range orientational order was confirmed by calculating the diffraction patterns and plotting projections along the 2-fold, 3-fold and 5-fold symmetry axes. The diffraction pattern was calculated as
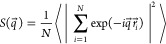
7where *q⃗* is the wave-vector. The diffraction pattern was averaged over 200 configurations.

The phason strain accounts for distortions of the quasiperiodic lattice in perpendicular space. Here, it was estimated from the slope of the distance between two particles in perpendicular space as a function of distance in parallel space.^6,20^ The positions of the particles were mapped onto lattice points in the 6D space using the lifting procedure described in the Supporting Information.

The simulation and all the analysis codes are available at (https://github.com/evanoya/MC_GPU).

## Supporting Information Available

Model design, methods and analysis of the assembled quasi-crystals (PDF)
Snapshots of particles (ZIP)
Nucleation and growth of the FCI QCs at (ϵ_BB_/ϵ_AA_ = 1.2) the transition from a gas of particles to a gas of icosahedra in the fluid phase (AVI)
Nucleation and growth of the FCI QCs above (ϵ_BB_/ϵ_AA_ = 1.4) the transition from a gas of particles to a gas of icosahedra in the fluid phase (AVI)

## Supplementary Material

nn4c14885_si_002.zip

nn4c14885_si_003.avi

nn4c14885_si_004.avi

## Notes

This manuscript has also been submitted to the arXiv server: Noya, E.G.; Doye, J.P.K., A one-component patchy-particle icosahedral quasicrystal. 2024, arXiv. https://arxiv.org/abs/2407.17212 (July 24th, 2024).
