# Supplementary material for: A One-Component Patchy-Particle Icosahedral Quasicrystal
Source: ACS Nano. 2025 Apr 1;19(14):13714–22. doi: 10.1021/acsnano.4c14885 (PMC12004934; doi:10.1021/acsnano.4c14885)
Supplement: Supplementary file 1 — nn4c14885_si_001.pdf [file nn4c14885_si_001.pdf]

Supporting Information for: "A one-component patchy-particle icosahedral quasicrystal"

Eva G. Noya*,† and Jonathan P. K. Doye*,‡

†*Instituto de Química Física Blas Cabrera, Consejo Superior de Investigaciones Científicas, CSIC, Calle Serrano 119, 28006 Madrid, Spain*

‡*Physical and Theoretical Chemistry Laboratory, Department of Chemistry, University of Oxford, South Parks Road, Oxford, OX1 3QZ, United Kingdom*

E-mail: eva.noya@iqf.csic.es; jonathan.doye@chem.ox.ac.uk

S1 Model design and methods

S1.1 Environment analysis

The first step to design a model system is to analyse the local environments in the target FCI QC. As can be seen in the radial distribution function of the ideal FCI QC (Fig. S5A), the first and second neighbours give rise to two well-separated peaks. In the local environment analysis, only the first neighbours are considered. The thirteen local environments, with between zero and seven neighbours, found in the ideal FCI QC are reported in Table S1. The angles between bonds in each local environment, as well as the proportion in which each local environment appears are also provided. The local environments with two or more neighbours are also depicted in Fig 1D in the main text. As can be seen in Table S1, the most frequent environments in the ideal FCI QC are also quite common both in the trigonal 3/2 and 5/3 approximants. However, some of the less common environments in the ideal

Table S1: Local environments in the ideal FCI QC. The number of nearest neighbours is defined as the number of particles within a distance of $1.3\,\sigma$ of the bond distance (this cutoff provides a clear distinction between first and second coordination shells, see Fig. S5). The angles formed between a particle and its pairs of nearest neighbours are provided in the third column. The proportion with which each local environment appears in the ideal FCI QC is given in the fourth column. The environment labelled as 12b appears in the approximants but not in the QC.

Index	$n_{\rm neigh}$	Angles	FCI QC	3/2 approximant	5/3 approximant
1	0		0.013	3/288=0.010	16/1220=0.013
2	1		0.003	3/288=0.010	3/1220=0.002
3	2	108.00	0.004	–	–
4	3	108.00×2, 60.00	0.002	–	3/1220=0.002
5	3	108.00 ×3	0.007	1/288=0.003	7/1220=0.006
6	4	108.00×3, 60.00×3	0.001	–	–
7	4	144.00, 108.00×4, 60.00	0.004	3/288=0.010	6/1220=0.005
8	5	144.00×2, 108.00×6, 60.00×2	0.066	21/288=0.073	84/1220=0.069
9	5	108.00×5, 60.00×5	0.003	–	3/1220=0.002
10	5	144.00×2, 108.00×5, 60.00×3	0.003	–	24/1220=0.020
11	6	144.00×2, 108.00×7, 90.00, 60.00×5	0.003	3/288=0.010	6/1220=0.005
12	6	144.00×3, 108.00×7, 90.00, 60.00×4	0.205	57/288=0.198	243/1220=0.199
12b	6	144.00×3, 108.00×6, 90.00×3, 60.00×3	–	2/288=0.007	3/1220=0.002
13	7	144.00×4, 108.00×9, 90.00×2, 60.00×6	0.686	195/288=0.678	837/1220=0.686

FCI QC are not present in these approximants. Similarly, the six-coordinate environment encountered (although in a very low proportion) in the two approximants and labeled as 12b in Table S1, is not present in the ideal FCI QC.

S1.2 Particle designs

The thirteen local environments found in the ideal FCI QC display a common geometry. All can be represented by a patchy particle mimicking the highest coordinate environment, the one with seven neighbours, if we allow for the possibility that not all the patches always form bonds. Our first model, named 7P FCI, consists of only one particle type with seven patches pointing towards the first neighbours in this environment. The patch vectors, as well as their reference vectors and offset angles for the torsion term, are provided in Table S2. Patches are classified into two types: five patches of type A, which often form intra-icosahedral bonds, and two patches of type B, which are mostly involved in inter-icosahedral bonds. We explored the relative interaction strength of the patch-patch interactions $\epsilon_{BB}/\epsilon_{AA}$ (taking ϵ_{AA}=1 and $\epsilon_{AB} = (\epsilon_{AA} + \epsilon_{BB})/2$), finding that in our simulations assembly is only

successful when $\epsilon_{BB}/\epsilon_{AA} > 1.2$.

Aiming at finding a simpler model system in which all the patch-patch interactions are equal, we propose a second model in which particles carry five equivalent B patches (instead of only two), named as 10P FCI model. Due to geometric constraints the maximum number of bonds is still limited to seven (although eight weaker bonds can be also found in transient configurations). Owing to the increased configurational entropy associated with the bonding of type B patches, this model is able to assemble into an FCI QC when all patch-patch interactions are of equal strenght. The geometric properties of the patches for this model are given in Table S3.

The 10P model can be further simplified by replacing the five A patches by a single ring patch located at a cone angle $\mu_A = \arccos(\tau/\sqrt{4\tau+3})$ =1.017 rad= 58.28° with respect to one pole of the particle, and the five B patches by a second ring patch at a cone angle $\mu_B = \arccos(\tau/\sqrt{2+\tau})$=0.5536 rad= 31.17° with respect to the opposite pole. The rings are thus represented by two patches pointing at opposite poles of the spherical particles and the angular term in the potential is modified to:[1]

$$V_{\text{ang,ring}}(\hat{\mathbf{r}}_{ij}, \mathbf{\Omega}_i, \mathbf{\Omega}_j) = \exp\left(-\frac{(\theta_{\alpha ij} - \mu_\alpha)^2}{2\sigma^2_{\text{ang,ring}}}\right) \exp\left(-\frac{(\theta_{\beta ji} - \mu_\beta)^2}{2\sigma^2_{\text{ang,ring}}}\right). \tag{S1}$$

$\theta_{\alpha ij}$ is the angle between the patch vector $\hat{\mathbf{P}}_i^\alpha$, representing the patch α, and $\hat{\mathbf{r}}_{ij}$. $\sigma_{\text{ang,ring}}$ is a measure of the angular width of the ring patch. We set $\sigma_{\text{ang,ring}}$=0.15 rad to reduce the surface area covered by the patches and prevent the appearance of a liquid phase. The torsional dependent term was not included for particles with ring patches. This model is named 2R FCI.

These three particle designs are depicted in Fig. S1.

Table S2: Description of the geometry of the patchy particle used to assemble the 7P FCI QC. The positions of the patches on the particle surface are specified by the patch unit vectors. For each patch, the reference vector and offset angles used for evaluating the torsional interactions are provided. Patches are divided into two types: type A comprises patches 1 to 5 that often form intra-icosahedral bonds, and type B includes patches 6 and 7 that most commonly form inter-icosahedral bonds. All the patches can interact with any other patch. The strength of the intra-icosahedral bonds is set to 1 ($\epsilon_{AA} = 1$), and an arithmetic mixing rule is used to calculate the cross interactions between inter- and intra-icosahedral patches ($\epsilon_{AB} = \epsilon_{AA} + \epsilon_{BB})/2$); thus, there is only adjustable parameter $\epsilon_{BB}/\epsilon_{AA}$.

Patch number	Patch type	Patch vector	Reference vector	Offset angles
P^1	A	(0.5, 0.30901699, -0.80901699)	(0, 0.52573111, 0.85065081)	0, 180
P^2	A	(0.80901699, -0.5, -0.30901699)	(0, 0.52573111, 0.85065081)	0, 180
P^3	A	(0, -1, 0)	(0, 0.52573111, 0.85065081)	0, 180
P^4	A	(-0.80901699, -0.5, -0.30901699)	(0, 0.52573111, 0.85065081)	0, 180
P^5	A	(-0.5, 0.30901699, -0.80901699)	(0, 0.52573111, 0.85065081)	0, 180
P^6	B	(0, 0, 1)	(0.85065807, 0, 0.52573111)	0, 180
P^7	B	(0.30901733, 0.80901699, 0.5)	(0.85065807, 0, 0.52573111)	0, 180

Table S3: Description of the geometry of the patchy particle used to assemble the 10P FCI QC. The positions of the patches on the particle surface are specified by the patch unit vectors. For each patch, the reference vector and offset angles used for evaluating the torsional interactions are provided. Patches are divided in two types: type A comprises patches 1 to 5 and often form intra-icosahedral bonds, and type B includes patches 6 to 10 that most commonly form inter-icosahedral bonds. All the patches can interact with any other patch with the same strength, which is set $\epsilon_{AA} = \epsilon_{BB} = \epsilon_{AB} = 1$. The width of the patches is set to $\sigma_{ang} = 0.25$ rad when the torsional contribution to the energy is not included and to $\sigma_{ang} = 0.30$ rad for the model with torsions. Narrower patches are required in the model without torsions to avoid the presence of a liquid phase (see main text for a more detailed explanation).

Patch number	Patch type	Patch vector	Reference vector	Offset angles
P^1	A	(0.5, 0.30901699, -0.80901699)	(0, 0.52573111, 0.85065081)	0, 180
P^2	A	(0.80901699, -0.5, -0.30901699)	(0, 0.52573111, 0.85065081)	0, 180
P^3	A	(0, -1, 0)	(0, 0.52573111, 0.85065081)	0, 180
P^4	A	(-0.80901699, -0.5, -0.30901699)	(0, 0.52573111, 0.85065081)	0, 180
P^5	A	(-0.5, 0.30901699, -0.80901699)	(0, 0.52573111, 0.85065081)	0, 180
P^6	B	(0, 0, 1)	(0.85065807, 0, 0.52573111)	0, 180
P^7	B	(0.5, 0.30901733, 0.80901699)	(0.52573111, 0.85065807, 0)	0, 180
P^8	B	(-0.5, 0.30901733, 0.80901699)	(0, -0.52573111, 0.85065807)	0, 180
P^9	B	(0.30901733, 0.80901699, 0.5)	(-0.52573111, 0.85065807, 0)	0, 180
P^{10}	B	(-0.30901733, 0.80901699, 0.5)	(-0.85065807, 0, 0.52573111)	0, 180

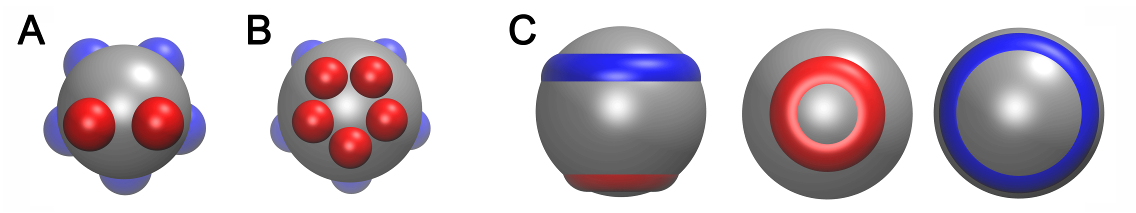

Figure S1: Particle designs studied in this work: (*A*) 7P , (*B*) 10P model and (*C*) 2R models (in *C*, from left to right: front, top and bottom views are depicted to ease visualization).

S1.3 Generalizability of approach

Here we have developed patchy-particle systems to form a FCI QC. This complements previous work that used as design targets IQCs that were generated from body-centred and primitive hypercubic lattices.[2] In these three cases, only the lattice points associated with the hypercubic lattices were projected onto the physical space. In principle, more target IQCs could be generated by projecting from hypercubic lattices using different occupation domains or with particles placed at additional positions in the hypercubic unit cell as well as the lattice points. How best to perform this in a way that generates target IQCs with inter-particle distances that can be realized with particles with well defined radii is less clear and a trial-and-error approach might be necessary. Experimental IQCs for which the structure has been determined could also be used as a source for target IQCs for our patchy-particle design procedure. However, patchy particles are generally less well-suited for realizing structures with high coordination numbers such as are typical in metallic alloy IQCs.

IQCs are the only QCs that are both quasiperiodic in all 3-dimensions and possess non-crystallographic symmetry. Our approach relies on the incompatibility of the particle's local patch geometry and interactions with any periodic crystalline symmetry to generate quasiperiodic order and so it is unlikely to be possible to use patchy particles to generate quasicrystals with crystallographic symmetries.[3]

Our general approach can also be used to generate patchy-particle systems that can form 3-dimensional axial QCs, i.e. QCs with quasiperiodic order in two dimensions and periodic order in the third. Examples with dodecagonal[4,5] and, very recently, octagonal[6] order have been reported. However, generating suitable target axial QCs is not straightforward, as although projection from higher-dimensional can be straightforwardly used to generate 2-dimensional quasiperiodic structures, how to best order the particles in the third dimension is less clear. Despite this, we hope that our general approach might not only be used to generate quasicrystals with all the symmetries observed experimentally, but also with symmetries not

yet realized in any material, e.g. a heptagonal QC.

S1.4 Lifting

"Lifting" involves mapping the positions of particles in an IQC configuration to lattice sites in a 6D hypercubic lattice. In effect it is the inverse of the cut-and-project method that was used to generate the ideal IQCs. The basic procedure we used is as follows. First, one starts at an arbitrary particle (we usually choose the particle closest to the centre of mass of a cluster) that is assigned to the origin of the 6D lattice. One then follows the bond network away from this particle assigning 6D lattice sites to its neighbours. This procedure is then iterated from the newly-assigned particles until all particles have been lifted.

To achieve this lifting one needs a mapping between a bond vector in a given direction and an inter-lattice vector in 6D. In our case the bonds are just along the 30 directions of the C_2 symmetry axes of the I_h point group. For example, vectors along the C_2 axis in the $+x$ direction can be generated by $\mathbf{b}_2 - \mathbf{b}_5$ (i.e. a sum of the five-fold vectors that pass through the vertices at either end of the edge of the icosahedron that the symmetry axis passes through) or $\mathbf{b}_3 + \mathbf{b}_6$ (i.e. a sum of the five-fold vectors that pass through the vertices at the opposite corners of the triangular faces containing the edge that the symmetry axis passes through), or any linear combinations thereof (Fig. S2). Note $|\mathbf{b}_2 - \mathbf{b}_5| = \tau|\mathbf{b}_3 + \mathbf{b}_6|$. Thus, in general, for a bond along the C_2 direction in x, the corresponding step in the 6-dimensional hypercubic lattice will be of the form:

$$\Delta\mathbf{l} = \frac{a}{2}\,(0, c, d, 0, -c, d) \tag{S2}$$

where c and d are integers. In our case, comparison of bonds in the ideal FCI QC with the lattice points from which they were projected allows us to determine values of c and d

consistent with this projection. Thus we obtain

$$\Delta\mathbf{l} = \frac{a}{2}\left(0, 2, 1, 0, -2, 1\right). \tag{S3}$$

Equivalent 6D displacement vectors for the other 29 bond directions can easily be derived. The length of the bond in the ideal FCI QC in terms of a is

$$r_0 = |\mathbf{Q}_{\rm par}\Delta\mathbf{l}^T| = \frac{1+2\tau}{\sqrt{4+2\tau}}a. \tag{S4}$$

If, as is the case for the current system, only bonds along the 2-fold axes are present, then the lifting is guaranteed to result in a face-centred hypercubic lattice. If the lattice sites are denoted by

$$\frac{a}{2}\left(n_1, n_2, n_3, n_4, n_5, n_6\right), \tag{S5}$$

where the n_i are integers, the definition of a face-centred lattice is that the $\sum n_i$ is even. It is easy to see that the change in this sum associated with the interlattice vector defined in Eq. S2 must also be even. Hence, lifting can only then generate lattice sites consistent with a face-centred lattice.

In practice, bonds in our simulated configurations will not exactly point along a given symmetry direction. We instead require that the dot product of the normalized bond vector with the unit vector along the symmetry direction must be greater than 0.98 to trigger lifting, adopting the convention that two particles are bonded if the distance between them is lower than 1.35 σ. Results are fairly similar for threshold values of the dot product within 0.97-0.99 and for bond cutoffs within 1.3-1.4 σ. We also exclude any particles from the analysis where ambiguous assignments arise due to multiple paths to the same particle leading to different assignments. In our case this is mainly due to some kind of local defect and the number of such instances is reduced with a stricter cutoff for lifting. If dislocations were present, the resulting ambiguity problem would effectively render the whole lifting process ill-defined.

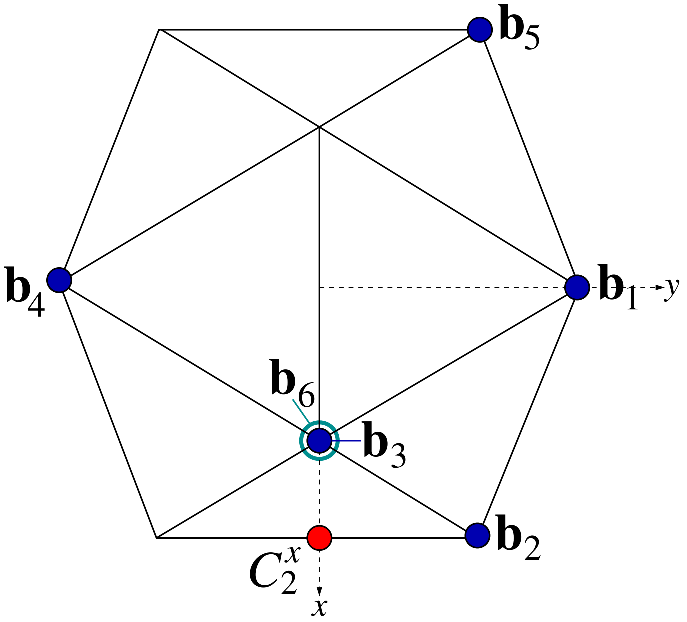

Figure S2: The $\mathbf{b}_i$ vectors obtained by projection of the unit vectors along the 6 dimensions of the hypercubic lattice into parallel space and their relationship to the five-fold symmetry axes of an icosahedron that is oriented with three of its two-fold symmetry axes along x, y and z. Note that the cyan ring associated with $\mathbf{b}_6$ is to signify it has a negative component in z.

Dislocations were not observed in any of our systems.

As expected, the lifting process only leads to lattice sites consistent with a face-centred lattice. We also checked the occupancy of the 32 lattice sites in the primitive face-centred unit cell. They had the same occupancy to within 0.1-0.2% further confirming the face-centred character of the assembled IQCs.

For the primitive (PI QC) and body-centred (BCI QC) icosahedral quasicrystals, the lifting procedure is analogous to the one just described. The main difference is that in the PI QC, bonds exist along the 2-fold and 3-fold axes, whereas in the BCI QC, bonds are present along the 2-fold, 3-fold, and 5-fold axes, with different bond lengths. In contrast, in the FCI QC all bonds are aligned with 2-fold axes. Given that the coordinates in 3D and 6D are known for the ideal QCs, mapping a bond in a given direction to an inter-lattice vector in 6D is straightforward.

S1.5 Indexing of diffraction patterns

The diffraction pattern along the five-fold, three-fold and two-fold symmetry axes of the ideal FCI QC and assembled QC are shown in Fig. S3. For comparison, projections of the pattern of the trigonal 3/2 approximant along equivalent axes are also shown. It can be observed that the brightest peaks in the pattern of the ideal structure are present in the diffraction pattern of the assembled QC, again supporting that it is of FCI type. This is more rigorously confirmed by indexing of the diffraction pattern.

Peaks in the IQC diffraction patterns are expected to occur at scattering vectors corresponding to the projection of the reciprocal lattice of the 6D hypercubic lattice onto parallel space. Six indices are required to index each peak. One common indexing scheme uses the six vectors obtained by projecting the six directions of the reciprocal lattice as a basis set; these vectors are along the fivefold axes of symmetry of I_h. We instead choose to use the indexing scheme where two basis vectors with a length ratio of τ along each of x, y and z are used; i.e.

$$\mathbf{q} = \frac{2\pi}{a\sqrt{4+2\tau}}(h+h'\tau, k+k'\tau, l+l'\tau). \tag{S6}$$

The peaks are then labelled using the notation $(h/h'k/k'l/l')$.

The 6D hypercubic lattice type can be identified from the diffraction pattern because each has a different set of extinction conditions. For an FCI system all indices must be even, and $h+l+h'+k'$ and $h+k+l'+k'$ (and $l+k+h'+l'$) must be a multiple of 4. The differences are most apparent for the diffraction pattern along the two-fold axis.

One of the difficulties of indexing IQC diffraction patterns is that the pattern of peaks is unchanged by a scaling of τ or τ^3 (depending on the symmetry direction). There is not simply a first peak that allows one to start the indexing and to work out the relationship between the real space distances and the hypercubic lattice parameter. Instead, when indexing experimental patterns the intensity of the peaks are often used to work out this relationship, as their intensity is related to the magnitude of $\mathbf{q}_{\text{perp}}$, where $\mathbf{q}_{\text{perp}}$ is the result of projecting

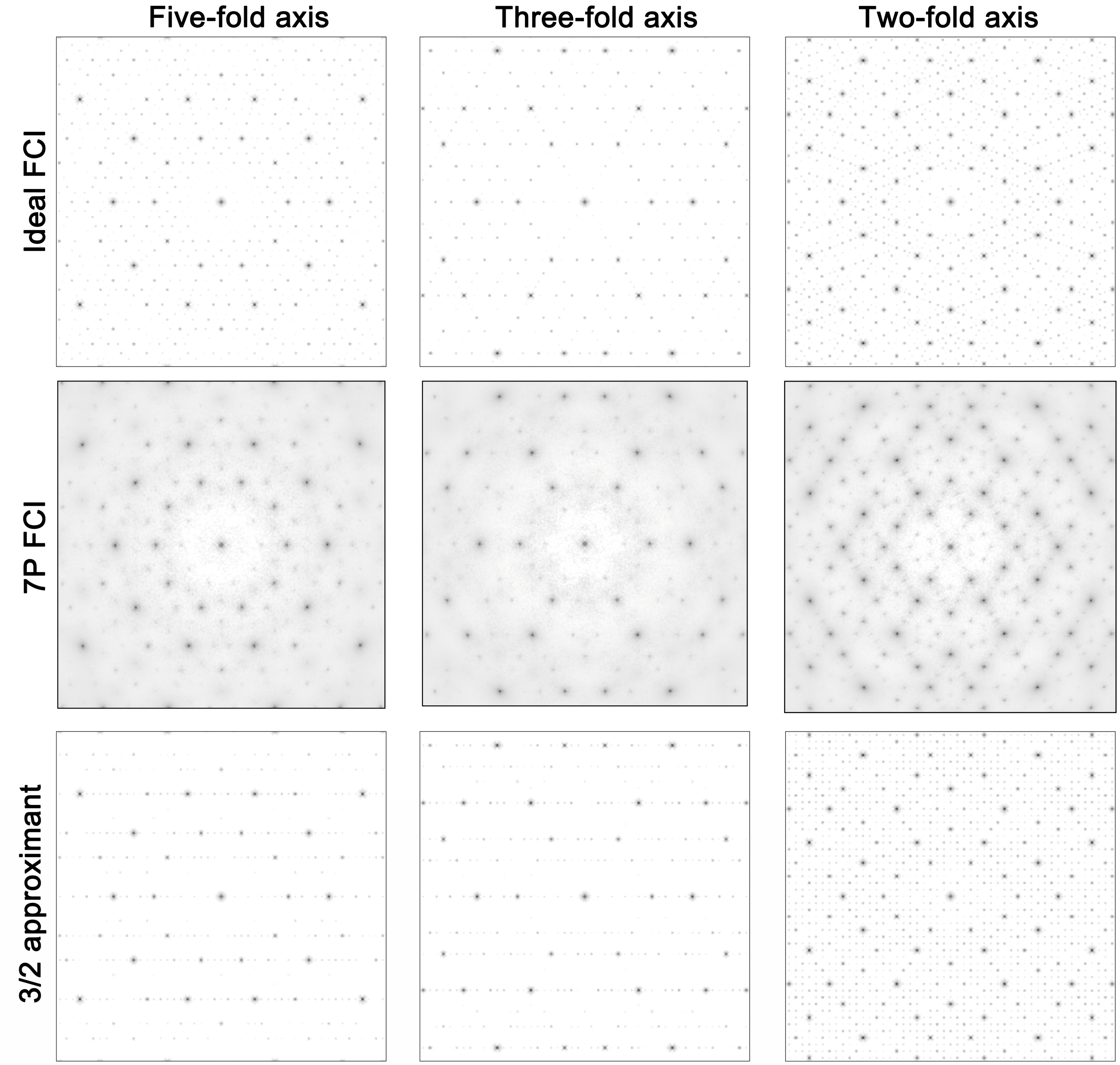

Figure S3: Diffraction pattern along the 5-fold, 3-fold and 2-fold symmetry axes of the ideal FCI QC, the assembled QC with the 7P model and $\epsilon_{BB}/\epsilon_{AA} = 1.2$. In the bottom row, projections of the diffraction pattern of the trigonal 3/2 approximant projected along the equivalent axes are also shown.

the relevant reciprocal lattice vector onto the perpendicular space.

$$\mathbf{q}_{\mathrm{perp}} = \frac{2\pi}{a\sqrt{4+2\tau}}[(h' - h\tau)\mathbf{i} + (k' - k\tau)\mathbf{j} + (l' - l\tau)\mathbf{k}] \tag{S7}$$

In particular, the intensity decreases with increasing q_{perp}.

In our case, however, we already know the relationship between the hypercubic lattice parameter and the bond distance in the ideal quasicrystal (Eq. S4), and so we can use this to index the peaks. For example, a peak at $(h/h', 0/0, 0/0)$ would be expected to occur on the x-axis at

$$|\mathbf{q}| = \frac{2\pi(h + h'\tau)(1 + 2\tau)}{(4 + 2\tau)r_0} \tag{S8}$$

where the average bond distance r_0 is expected to be approximately equal to $2^{1/6}\sigma_{\mathrm{LJ}}$, the position of the minimum in the Lennard-Jones potential.

A diffraction pattern along the 2-fold axis indexed in this manner is illustrated in Fig. S4. The pattern shows the distinctive features of an FCI QC with all observed peaks consistent with the extinction conditions for this lattice.

S2 Results

S2.1 Effect of the interaction strength

As discussed in the main text, simulations of the 7P FCI model lead to the assembly of the IQC for interaction strengths within $1.2 \lesssim \epsilon_{BB}/\epsilon_{AA} \lesssim 1.8$. As can be seen in Fig. S5, the radial distribution function and radial density are very similar for all the interaction strengths and also when a torsional term is added. Some differences are found in the distribution of the coordination numbers (Fig. S5C). All the assembled clusters exhibit a lower proportion of 7-coordinate particles than the ideal FCI QC. In the ideal FCI QC about 65% of the particles are fully bonded, whereas in the assembled QC this percentage falls down to 45-60%. The highest percentage is attained for $\epsilon_{BB}/\epsilon_{AA} = 1.2$, and it decreases either when $\epsilon_{BB}/\epsilon_{AA}$

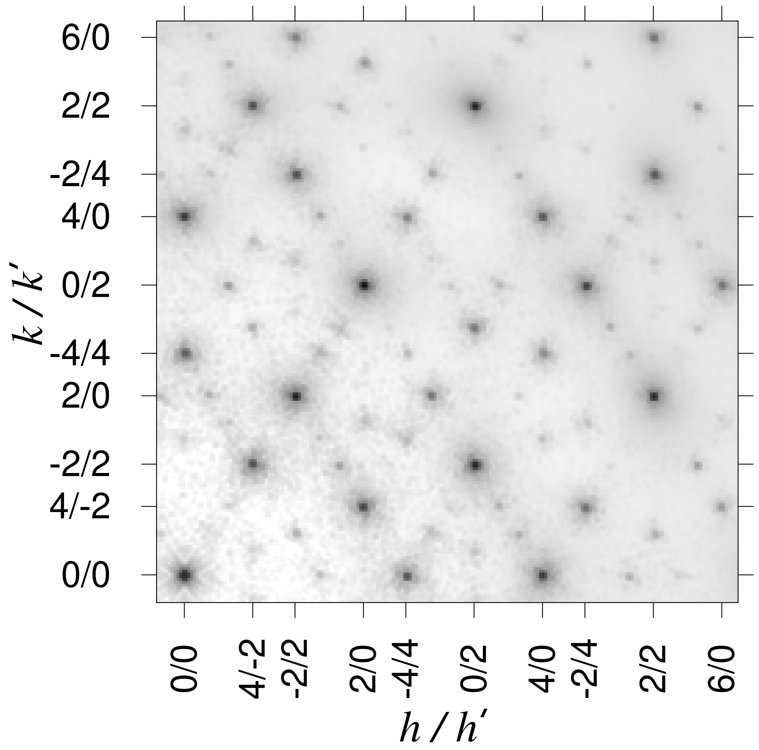

Figure S4: Indexed diffraction pattern for the assembled IQC viewed along a two-fold axis.

increases and when torsions are included. The average coordination number (Table S4) is also similar in all the systems, but again the system with $\epsilon_{BB}/\epsilon_{AA} = 1.2$ is the one with an average coordination closer to that of the ideal FCI QC. Projections of the diffraction pattern along the symmetry axes (Fig. S6) are very similar independent of the model parameters or the presence of the torsional term. Some differences appear in the phason strain plots (Fig. S5D), with higher strains appearing for higher values of $\epsilon_{BB}/\epsilon_{AA}$ and when the potential contains the torsional term (Table S5).

S2.2 Structure of the fluid before nucleation

With the aim of gaining insight into the mechanism of assembly, we analysed the structure of the fluid before nucleation in some of the model systems. Representative configurations of the fluid and the cluster size distribution (CSD) are presented in Fig. S8. As expected, the CSD shows that larger clusters become more frequent as $\epsilon_{BB}/\epsilon_{AA}$ increases. Inspection

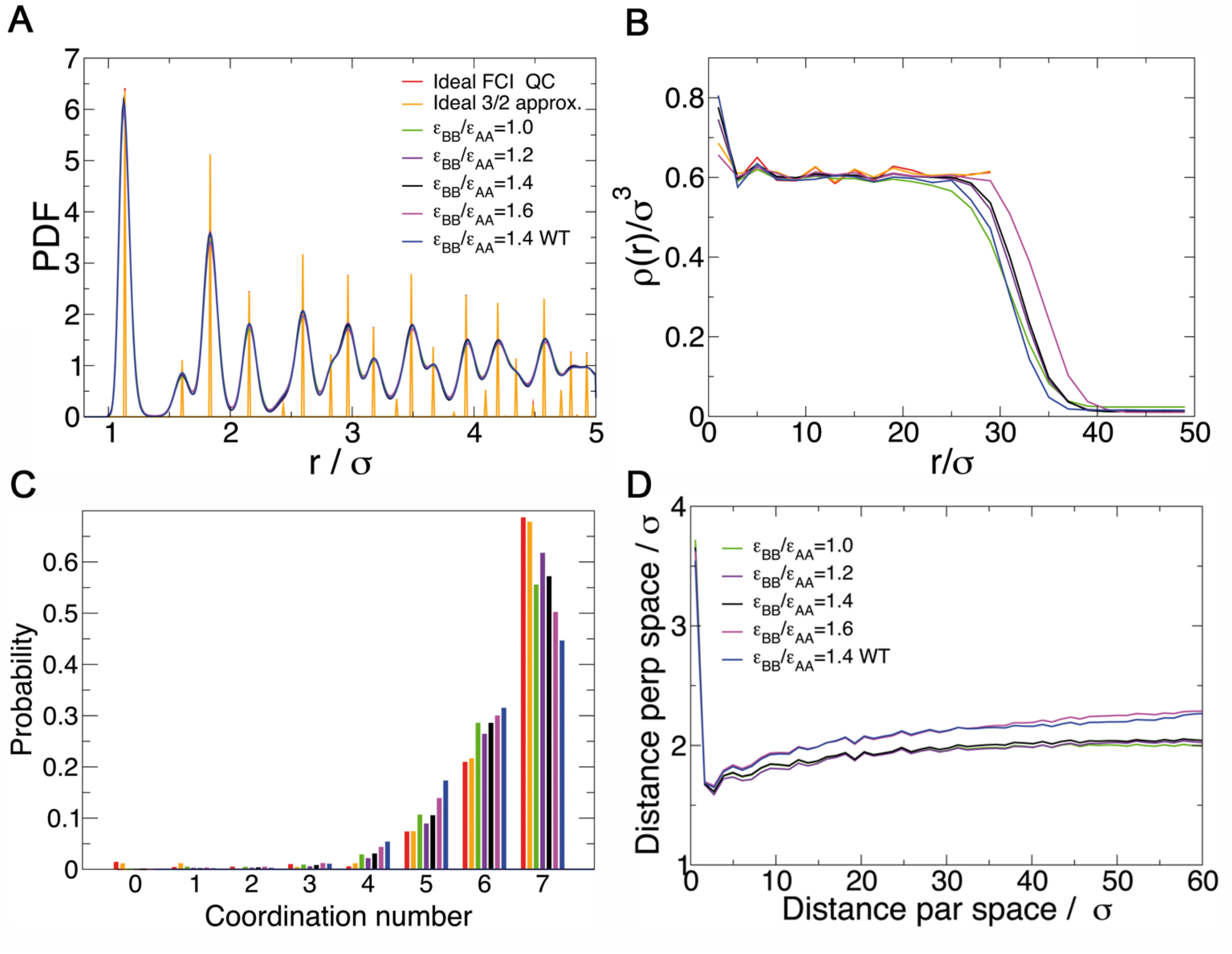

Figure S5: (*A*) Pair distribution function, (*B*) radial density, (*C*) coordination number and (*D*) phason strain of the assembled QC with the 7P FCI model at different $\epsilon_{BB}/\epsilon_{AA}$ interaction strengths and with and without torsions, as indicated in the legends. The temperatures at which each model was simulated are given in Table S4. Assembly of the IQC from the gas phase was not observed in our simulations of the 7P FCI model with $\epsilon_{BB}/\epsilon_{AA} = 1$. For this case, these structural properties were evaluated in a cluster grown with $\epsilon_{BB}/\epsilon_{AA} = 1.2$, subsequently simulated with $\epsilon_{BB}/\epsilon_{AA} = 1.0$. For comparison, results for the ideal FCI QC and its 3/2 approximant (without zero-coordinated particles) are also provided. The peaks of the PDF of the ideal structures have been scaled to ease comparison with the simulated PDFs. The ideal FCI QC and its trigonal 3/2 approximant exhibit very similar PDFs within the shown distance range, with peaks exactly at the same distances and only small differences in the peaks intensity.

Table S4: Fraction of AA, BB and AB bonds (x_{AA}, x_{BB} and x_{AB}) and average coordination number ($\langle CN \rangle$) in the simulated IQCs with the 7P and 10P FCI models, along with the simulated temperature and the equilibrium cluster size (N_c). Results for the ideal FCI QC and the trigonal 3/2 approximant simulated with the 7P FCI model and $\epsilon_{BB}/\epsilon_{AA} = 1.0$ are also provided. Two particles are considered bonded if the interaction between them is lower than -0.20 ϵ. For a few cases, the average coordination number was also calculated using a distance criteria with a cutoff distance of 1.3 σ to define first neighbours (data within parentheses). Typically only those particles within a sphere of radius 20 σ from the center of mass, which are sufficiently away from the cluster surface (see Fig. S5B), are considered in this analysis. For comparison, the average coordination number in the ideal FCI QC and the lower approximants are given in the lower rows.

System	T^*	N_c	x_{AA}	x_{BB}	x_{AB}	$\langle CN \rangle$
7P, $\sigma = 0.30$, ϵ_{BB} =1.0, NT	0.154	80000	0.65	0.21	0.14	6.33
7P, $\sigma = 0.30$, ϵ_{BB} =1.2, NT	0.159	69000	0.65	0.22	0.13	6.43 (6.51)
7P, $\sigma = 0.30$, ϵ_{BB} =1.4, NT	0.164	85000	0.63	0.22	0.15	6.37
7P, $\sigma = 0.30$, ϵ_{BB} =1.6, NT	0.170	100000	0.60	0.22	0.19	6.20
7P, $\sigma = 0.30$, ϵ_{BB} =1.4, WT	0.159	75000	0.60	0.19	0.21	6.10
Ideal FCI QC, 7P, $\sigma = 0.30$, ϵ_{BB} =1.0, NT	0.154	82000	0.65	0.23	0.12	6.44
Ideal 3/2 AC, 7P, $\sigma = 0.30$, ϵ_{BB} =1.0 NT	0.154	82000	0.65	0.23	0.12	6.44
10P $\sigma = 0.25$, $\epsilon_{BB} = 1$, NT	0.151	77000	0.62	0.22	0.16	6.42 (6.58)
10P $\sigma = 0.30$, $\epsilon_{BB} = 1$, WT	0.154	90000	0.56	0.20	0.24	6.25 (6.59)
2R $\sigma = 0.15$, $\epsilon_{BB} = 1$	0.166	83000	0.61	0.25	0.14	6.70 (7.37)
0/1 approximant	–	–	–	–	–	4.0
1/1 approximant	–	–	–	–	–	6.38
2/1 approximant	–	–	–	–	–	6.44
3/2 approximant	–	–	–	–	–	6.45
5/3 approximant	–	–	–	–	–	6.46
8/5 approximant	–	–	–	–	–	6.46
FCI QC	–	–	–	–	–	6.47

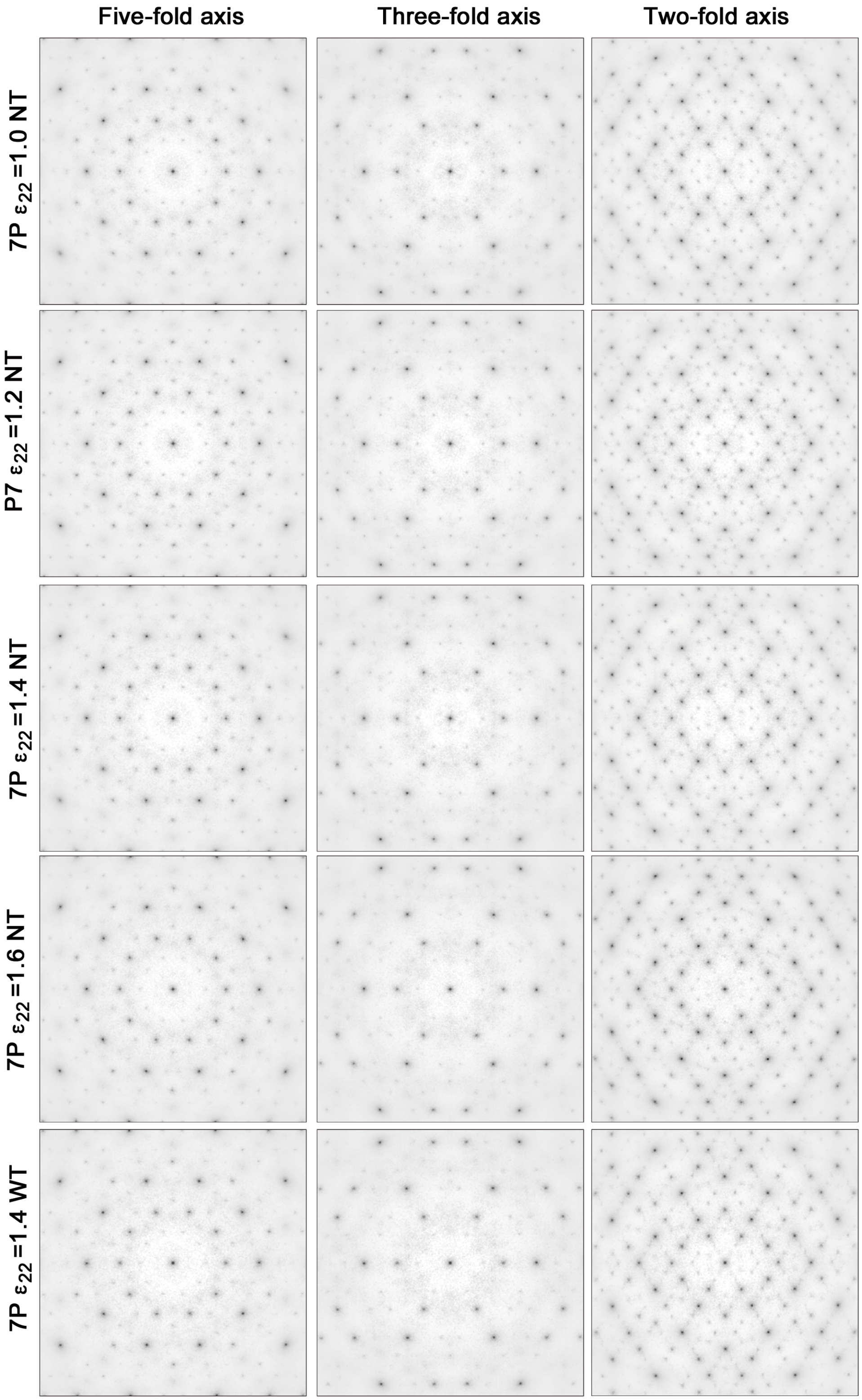

Figure S6: Diffraction pattern of the assembled IQC with the 7P FCI model for different $\epsilon_{BB}/\epsilon_{AA}$ interaction strengths and with and without torsions. The temperatures at which each model was simulated are given in Table S4.

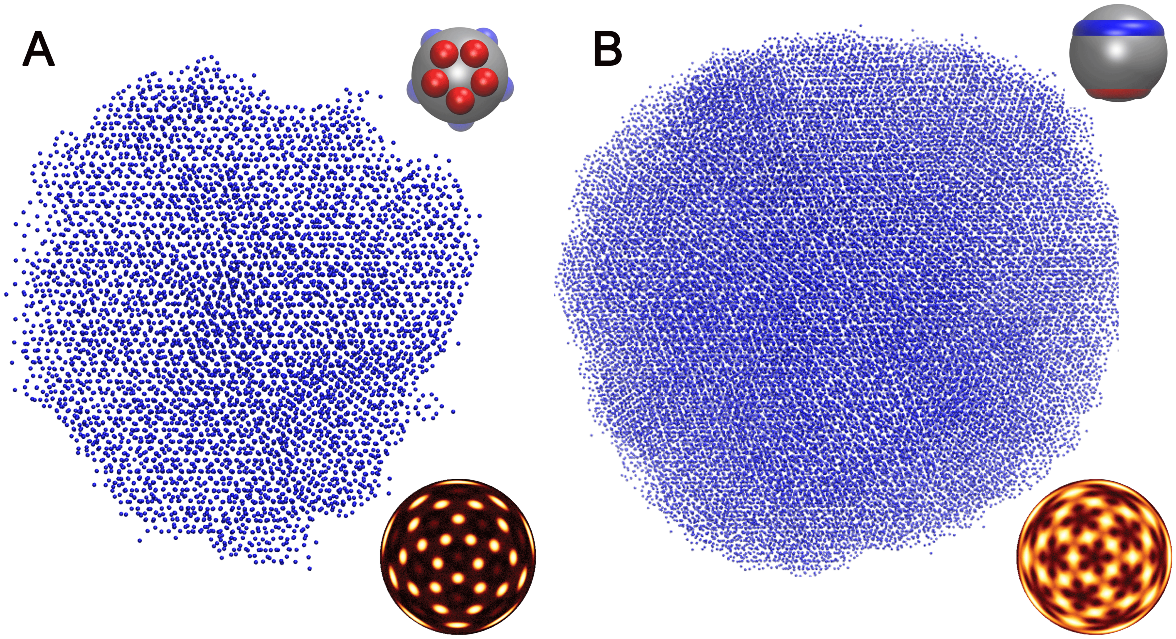

Figure S7: Snapshots of (*A*) a 17 000 particle FCI QC cluster assembled from a gas of 10P particles and (*B*) a 83 000 particle FCI QC cluster assembled from a gas of 2R particles, shown in the top right corners. Particles in the clusters are displayed as small spheres. In these representations the global five-fold order is apparent as well as the presence of phason disorder but the absence of dislocations (see also Fig. S14). As can be seen in the BOOD, bonds are again aligned along the two-fold axes, although they are less constrained in the 2R system. The diffraction patterns for these IQC are shown in Supplementary Fig. S10.

Table S5: Phason strain measured as the slope of the distance in perpendicular space between two lifted lattice points as a function of their separation in parallel space (Figs. 3J, S5D, S10F and S11I). Fits were measured considering only distances where the slope of the graph was approximately constant, namely between 40-60 σ in parallel space, except for the 10P and 2R models, for which parallel distances between 30-50 σ were considered. Phason strain in the 7P model with $\epsilon_{BB}/\epsilon_{AA}$ =1, is about 100 times lower than in the 3/2 approximant and approximately of the same order as that in the annealed ideal FCI QC with the same model. We can conclude that this system has zero phason strain within the accuracy of our calculations. The last column shows the percentage of lifted particles that fall outside the OD, which is set to a triacontrahedron with an inscribed sphere having a radius equal to 2σ.

System	Phason strain	Points outside OD
7P, $\sigma = 0.30$, ϵ_{BB} =1.0, NT	0.0002	8%
7P, $\sigma = 0.30$, ϵ_{BB} =1.2, NT	0.002	6%
7P, $\sigma = 0.30$, ϵ_{BB} =1.4, NT	0.001	8%
7P, $\sigma = 0.30$, ϵ_{BB} =1.6, NT	0.004	6%
7P, $\sigma = 0.30$, ϵ_{BB} =1.4, WT	0.005	11%
Ideal FCI QC, 7P, $\sigma = 0.30$, ϵ_{BB} =1.0, NT	0.0001	0%
Ideal 3/2 AC, 7P, $\sigma = 0.30$, ϵ_{BB} =1.0 NT	0.02	0.004%
10P $\sigma = 0.25$, $\epsilon_{BB} = 1$, NT	0.004	5%
10P $\sigma = 0.30$, $\epsilon_{BB} = 1$, WT	0.002	7%
2R $\sigma = 0.15$, $\epsilon_{BB} = 1$, NT	0.005	13%

of the configurations reveals that these large clusters are disordered, and that icosahedra become more frequent as $\epsilon_{BB}/\epsilon_{AA}$ decreases. The presence of icosahedra in the gas phase significantly slows down the growth rate of the cluster (Fig. S8B). The 7P FCI model with $\epsilon_{BB}/\epsilon_{AA} = 1.2$ is the model with lowest growth rate. It is plausible that this slow growth provides the required time for defects to anneal out, which may appear when particles in the gas phase adhere to the growing cluster. Indeed, the assembled IQC with this model is the one structurally most similar to the ideal FCI QC and with lowest phason strain (Table S5).

S2.3 Estimation of the transition from a gas of particles to a gas of icosahedra

The transition from a gas of monomers to a gas of icosahedra was investigated by conducting simulations of particles with only five patches of type A, i.e. the ones involved in intra-icosahedral bonds, thus, allowing us to study this transition unhindered by further assembly of the icosahedra into higher-order structures mediated by the B patches. This makes the particles equivalent to those previously studied in Refs. 1,7. The patch width was set to $\sigma_{ang} = 0.3$ radians, as in the 7P FCI model. Monte Carlo simulations were performed in a cubic simulation box with 20 000 particles at a density of $0.1\,\sigma_{LJ}^{-3}$ and at three different temperatures, starting from a random configuration of a gas of monomers. Simulations were run for about 20–30 million MC cycles until equilibrium was reached. The proportion of clusters of sizes between 1 and 12 was monitored for 10 additional million MC cycles. To double-check convergence, separate simulations were run at $T^* = 0.158$ starting from a gas of monomers and a gas of mostly icosahedra; the results were effectively identical.

The centre of the transition from a gas of monomers to a gas of icosahedral clusters is estimated to occur at $T^* = 0.153$ and is where 50% of particles are in icosahedra. This result is lower than the value of T^*=0.156 that was obtained by umbrella sampling Monte Carlo simulations of 5-patch particles in a system containing 12 particles;[1] however, that study neglected finite-size effects,[8] whereas for the large systems used here these effects are

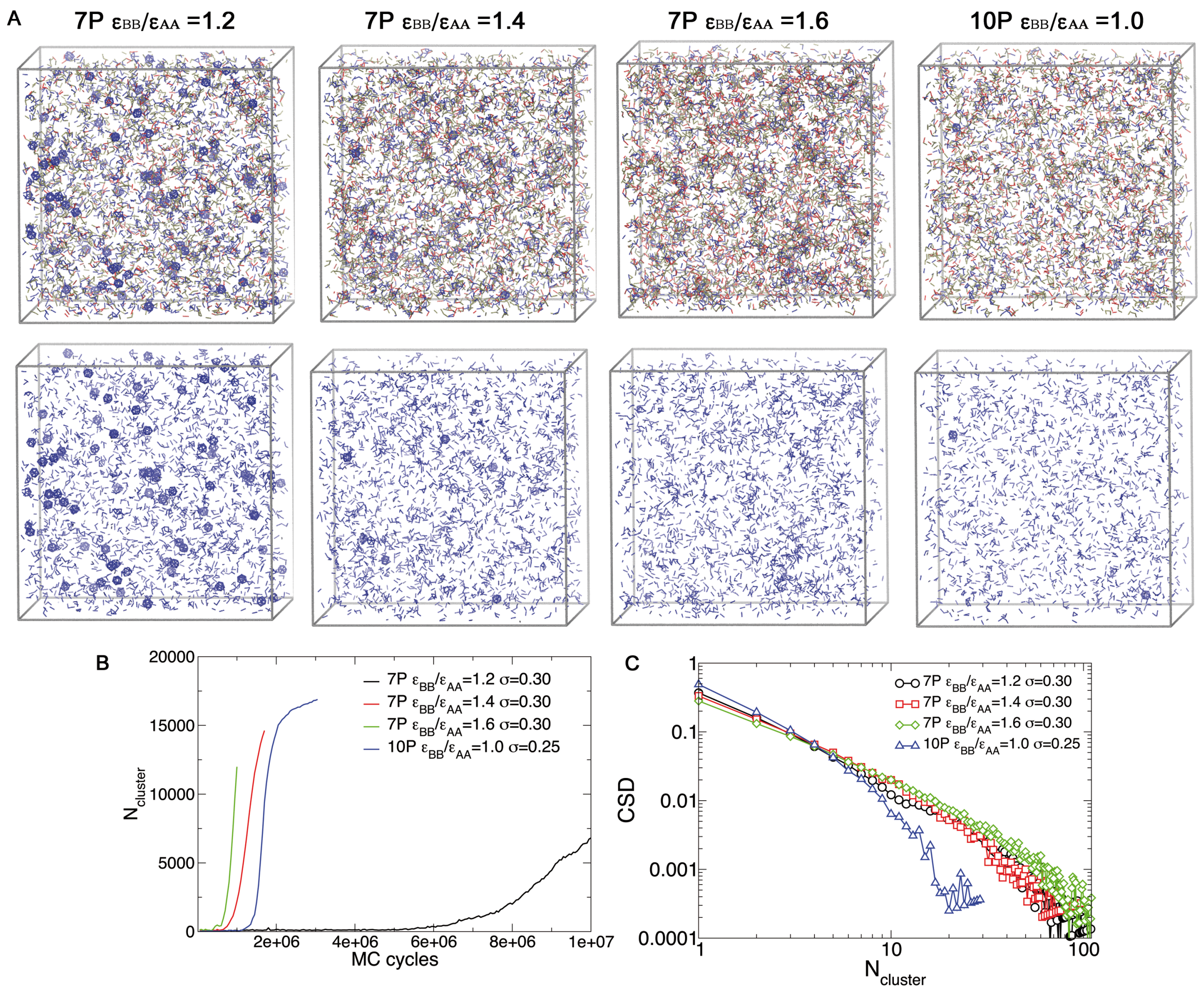

Figure S8: (A) Representative snapshots of the fluid ($\rho^* = 0.1$) before nucleation of the IQC in the 7P and 10P systems. In the top row, blue lines represent AA (intra-icosahedral) bonds, red lines BB (inter-icosahedral) bonds and tan lines AB bonds. In the bottom row only the AA bonds are shown to aid visualization. The number of icosahedra in the 7P FCI model decreases as $\epsilon_{BB}/\epsilon_{AA}$ increases. Some icosahedra are visible in the 10P system, in a proportion that is intermediate between those of the 7P system with $\epsilon_{BB}/\epsilon_{AA}$=1.4 and $\epsilon_{BB}/\epsilon_{AA}$=1.6. ($B$) Growth rate of the FCI QC cluster from the gas phase. (C) Normalized cluster size distribution (CSD) in the fluid before the nucleation of the IQC. The CDS gives the the fraction of particles belonging to a cluster of size N_{cluster}. In the 10P system large clusters (above 30 particles) are less frequent than in the 7P system (for which clusters of up to 100 particles are observed for the three values of $\epsilon_{BB}/\epsilon_{AA}$). For all the model systems, data corresponds to the highest temperature at which nucleation was observed on the time scale of our simulations.

negligible. The temperatures at which 70% ($T^* = 0.149$) and 30% ($T^* = 0.157$) of particles assemble into icosahedra provides an indicator of the width of the transition (see Fig S9).

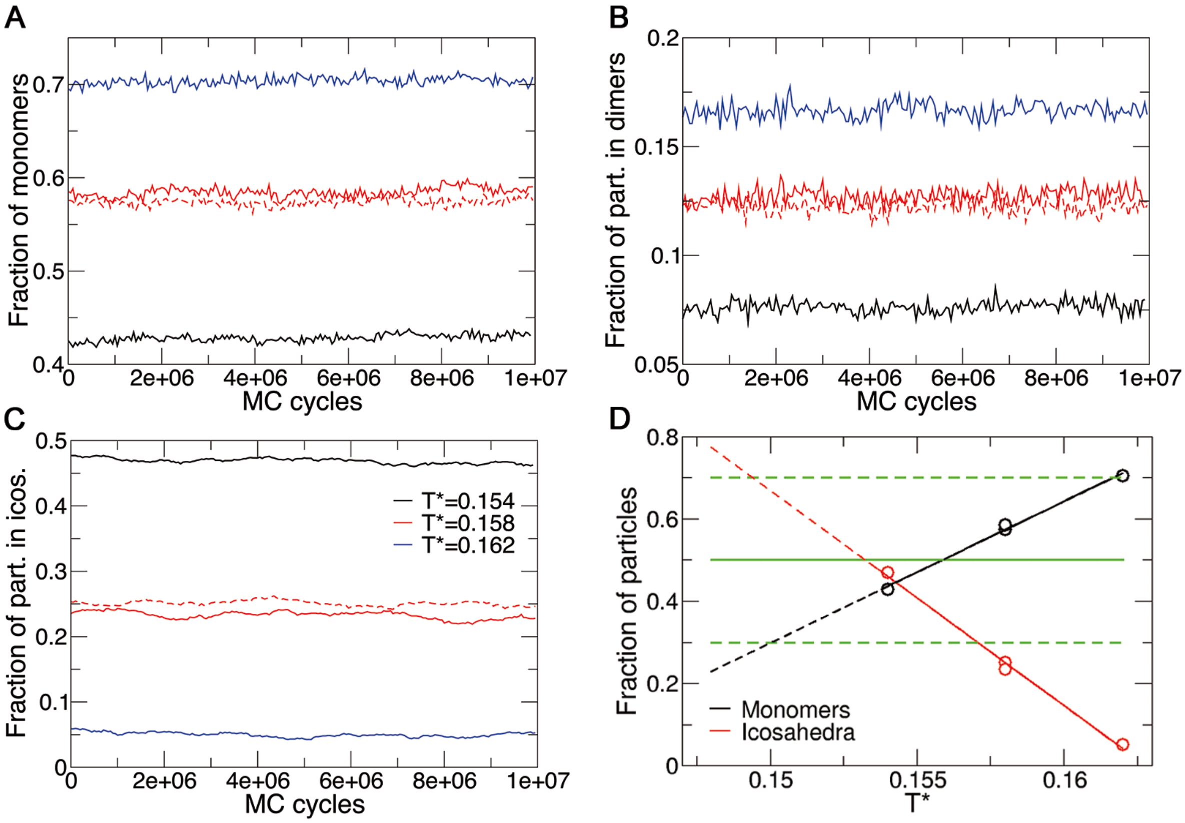

Figure S9: (A)-(C) Evolution of the fraction of particles belonging to (A) monomers, (B) dimers and (C) icosahedra at T^*=0.154, 0.158 and 0.162. At T^* =0.158 two trajectories are shown: the straight line corresponds to a simulation starting from a random disordered configuration and the dashed line was obtained starting from an equilibrated configuration at $T^* = 0.154$, in which a large proportion of particles are forming icosahedra. (D) Fraction of particles belonging to monomers and to icosahedra as a function of temperature. The lines are linear fits. The horizontal green solid and dashed lines mark the 0.5 and 0.5±0.2 probabilities. The transition temperature from a gas of particles to a gas of icosahedra was taken as that at which a 0.5 fraction of particles are forming icosahedra.

S2.4 Nucleation and growth mechanisms

Although conducting a rigorous study on the nucleation and growth mechanisms of the FCI QC is beyond the scope of this work, some insights can already be gained from a visual inspection of the trajectories. Two different nucleation and growth mechanisms were

observed at ($\epsilon_{BB}/\epsilon_{AA} = 1.2$) and above ($\epsilon_{BB}/\epsilon_{AA} = 1.4$) the transition from a gas of particles to a gas of icosahedra of the fluid. These are shown in the Supplementary Movies M1 and M2. The movies correspond to the trajectories shown in Fig. S8. As can be seen, the growth rate is significantly slower for the model with $\epsilon_{BB}/\epsilon_{AA} = 1.2$ than for that with $\epsilon_{BB}/\epsilon_{AA} = 1.4$. The movies show the evolution of the largest cluster from its birth until it reaches a fairly large size. For clarity, only bonds (not particles) are shown. These are depicted in different colours depending on the assembled state of each particle: particles in the largest solid cluster identified with an order parameter (similar to that used by Keys and Glotzer,[9] but with $q_{12}(i)q_{12}(j)$ as this exhibits a distinctive signal for the IQC) are shown in blue, those bonded to the largest cluster but not identified as quasicrystalline are shown in purple, and those pertaining to clusters of bonded particles with sizes larger than 10 particles are shown in green.

As can be seen in the Supplementary Movie M1, for the system with $\epsilon_{BB}/\epsilon_{AA} = 1.2$, many icosahedra are present in the fluid phase before nucleation. Some of these icosahedra bind to each other, initially not necessarily with the right orientation or arrangement of B patches on their surface, but as the simulation progresses, they are able to reorient and rearrange and thus promote growth. This is a rather slow process, and several crystallites compete with each other. Eventually, one of the crystallites outweighs the others and continues to grow by attachment of further icosahedra from the fluid phase. There are also a significant number of particles bonded to the surface of the solid cluster which are not part of complete icosahedra and are likely also contributing to cluster growth. Overall, we can conclude that in this case, cluster growth is primarily driven by the addition of icosahedra from the fluid phase. By contrast at $\epsilon_{BB}/\epsilon_{AA} = 1.0$, the icosahedra are insufficiently dynamic to allow growth of a large IQC.

The mechanism in the system with $\epsilon_{BB}/\epsilon_{AA} = 1.4$ is notably different (Movie S2). In this case, there are far fewer icosahedra in the fluid phase before nucleation. Particles still tend to form relatively large aggregates, but these are now more disordered, with only a

few icosahedra present. In this example, growth begins from a dimer of icosahedra, which, over time, grows primarily through single-particle addition. However, the addition of a few icosahedra is also observed during cluster growth. Single particles can align more quickly and coherently with the orientational symmetry of the formed cluster than the icosahedra, which is why cluster growth is significantly faster in this system than in the previous one.

S2.5 Comparison of the 7P, 10P and 2R FCI models

The structural properties of the assembled IQCs with the 7P, 10P and 2R FCI models are compared in Fig. S10. The 7P and 10P models form structurally very similar IQCs. The brightest peaks in the diffraction pattern are shared by these two model systems. The PDFs are also very similar. The only appreciable difference is that peaks in the 10P FCI system with $\sigma_{ang} = 0.25$ radians are more defined, which is attributed to the narrower patch width. The density of the 10P IQCs is slightly larger than that of the 7P IQC, which can be attributed to the appreciable number of particles with eight nearest neighbours in the 10P system (Fig. S10E). If all neighbours are located at the minimum energy position, only seven nearest neighbours are possible due to geometric constrains. However, up to eight bonds are possible if the neighbour particles are slightly displaced from the energy minimum positions (Fig. S10G). This comes with an energy penalty and, indeed, when coordination numbers are calculated using an energy criterion 7P and 10P IQCs exhibit similar average coordination numbers (Table S4), but applying a distance criterion $\langle CN \rangle$ is about 1% lower in the 10P IQCs.

When the two sets of patches are replaced by two ring patches, the diffraction pattern again exhibits the main features of the 7P and 10P, although there might be some changes in the peaks' relative intensities. Some small differences are also visible in the PDF. The peaks become somewhat lower and broader in the 2R QC. The radial density is about 2-3% higher in this case, which might be related to a significant number of particles with eight neighbours (about 20% have coordination eight, Fig. S10 *E*). The BOOD is also very different from

those in the 7P and 10P QCs. The bright spots become much broader, indicating that bonds are more loosely constrained to the 30 directions of the C_2 symmetry axes of the I_h point group than in the 7P and 10P QCs. The phason strain is somewhat larger than to those of the 7P and 10P models (Table S5), but the number of unassigned particles due to conflicting assignments is significantly larger than in the remaining particle designs (about 10% cannot be lifted, as compared to less than 1% in the 7P and 10P systems). This is likely due to the greater disorder in this system.

S2.6 Simulations of the ideal FCI and 3/2 approximant with the 7P model

A complete structural and dynamic characterization of the annealed ideal FCI QC and the 3/2 approximant simulated with the 7P particle design with all patches having equal interaction strength $\epsilon_{BB}/\epsilon_{AA} = 1$ is provided in Fig. S11.

S2.7 Particle mobility during the simulations: The evolution of the Van Hove correlation function

The mobility of the particles along the simulations was quantified by evaluating the Van Hove correlation function, measured after 1 million MC cycles in the 7P FCI system with $\epsilon_{BB}/\epsilon_{AA} = 1.4$. Evaluation of the correlation at different stages of a long annealing simulation reveals that particle mobility is higher at the early stages of the simulation, but then reaches a steady state of lower mobility (Fig S13); this change is probably due to the annealing out of defects. Note however that probabilities of particle hops after 1 million MC cycles are quite small and do not lead to significant relaxation of the structure, even after the 10 million long simulation. Both the radial density and energy and the phason strain are almost indistinguishable at the beginning and at the end of the simulation (Fig S13).

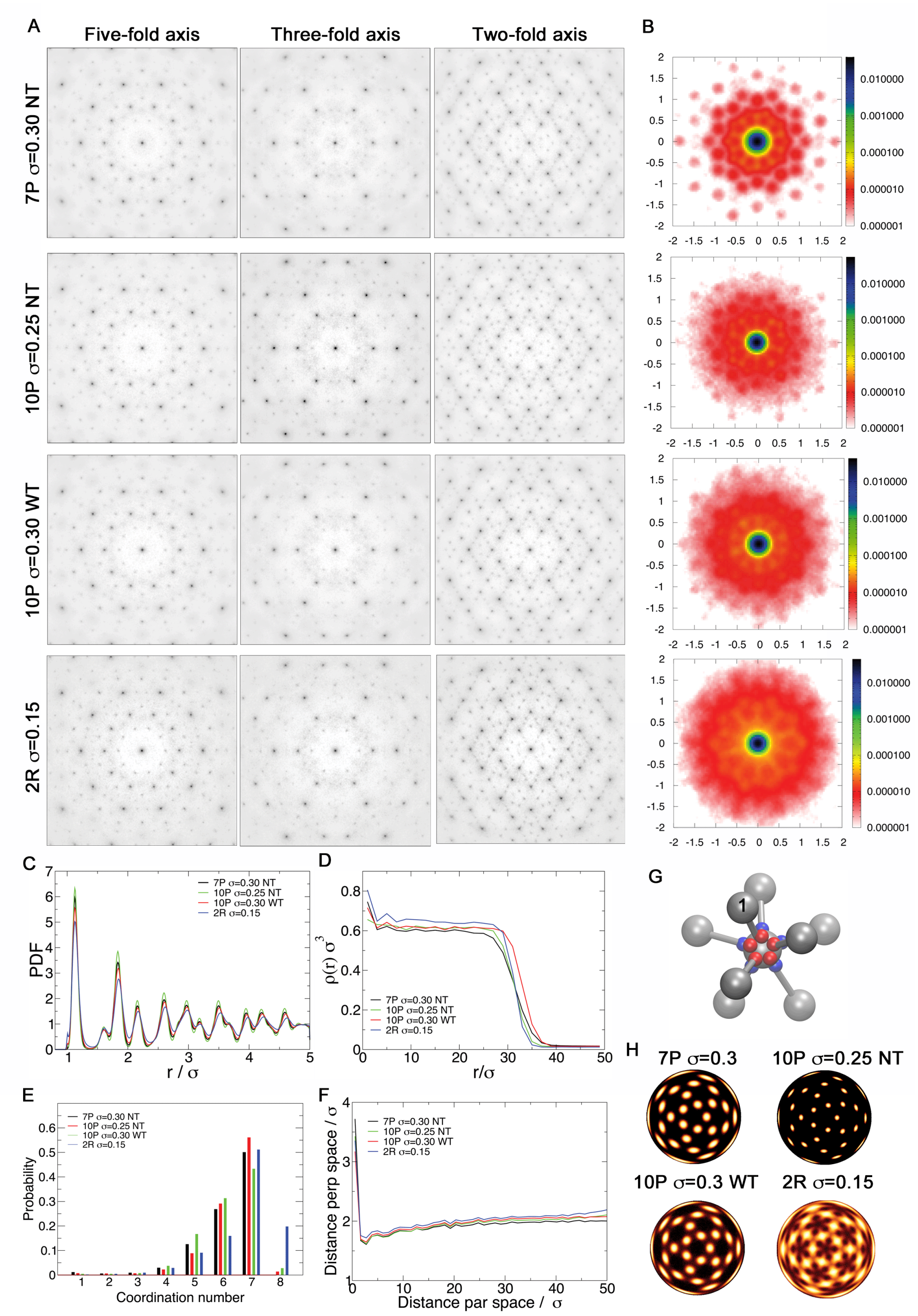

Figure S10: Comparison of the IQC assembled with the 7P, 10P and 2R models. (*A*) Projections of the diffraction pattern along 5-fold, 3-fold and 2-fold axes. (*B*) Van Hove correlation function. (*C*) Pair distribution function. (*D*) Radial density. (*E*) Coordination number distribution. (*F*) Phason strain. (*G*) An example particle bonded to 8 neighbours extracted from the 10P $\sigma = 0.25$ NT system. The Particle with the label "1" is able to squeeze inbetween two particles bonded to the B patches to form an extra weak bond, in this particular example the energy of the bond is $-0.40\,\epsilon$. We speculate that these defects help to make the 10P QC more 'dynamic', as shown by the Van Hove correlation plots. (*H*) BOOD diagrams. Results for the 7P FCI model correspond to $\epsilon_{BB}/\epsilon_{AA} = 1.2$.

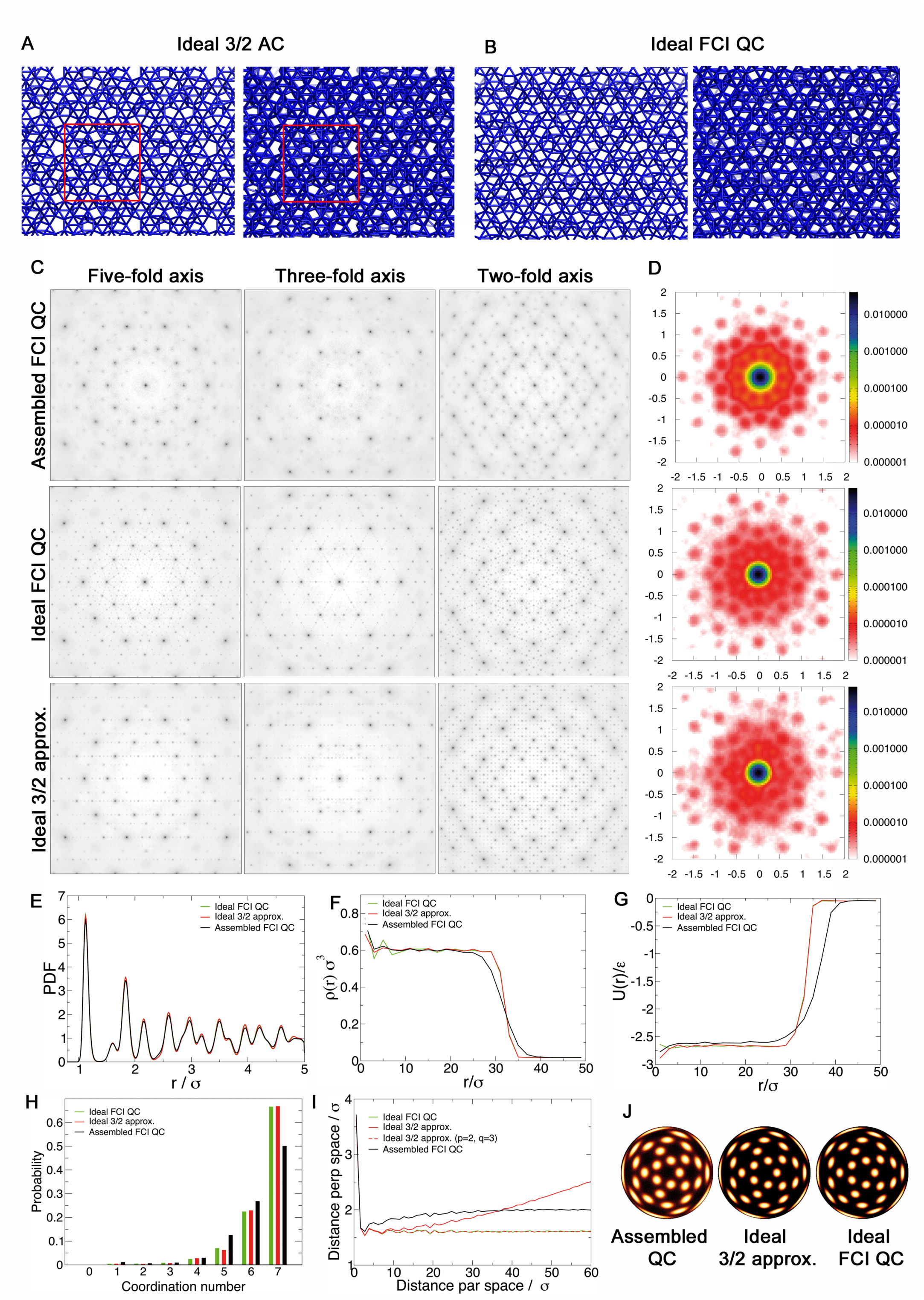

Figure S11: Annealed ideal FCI QC and a 3/2 approximant versus the assembled IQCs. The three systems were simulated using the 7P design with $\epsilon_{BB}/\epsilon_{AA} = 1$ at $T^* = 0.154$. Structure of the pristine and tempered (*A*) 3/2 AC and (*B*) ideal FCI QC. (*C*) Projections of the diffraction pattern along 5-fold, 3-fold and 2-fold axes. (*D*) Van Hove correlation function. (*E*) Pair distribution function. (*F*) Radial density. (*G*) Radial energy. (*H*) Coordination number distribution. (*I*) Phason strain. (*J*) BOOD diagrams.

S2.8 Further results of lifting

Additional information about the occupation domain (OD) of the ideal FCI and 3/2 approximants as well as that of the assembled QCs obtained from the lifting procedure is provided in Fig. S15. For completeness, we have also applied the lifting analysis to the primitive (PI QC) and body-centred (BCI QC) icosahedral quasicyrstals obtained in our previous work[2] (Fig. S16). The results are somewhat similar to those of the FCI QCs. Most of the assembled QCs exhibit very low phason strain, except for the BCI 3P and PI 2P systems. As in the FCI QCs, the energy of the particles is again rather independent of their distance from the center of the occupation domain. Curiously, two clearly distinct regimes can be observed in the plots of percentage of points outside the OD as a function of the radius of the inscribed sphere for the assembled PI QCs (Fig. S16F): there is an initial sharp drop coinciding with that of the ideal PI QC, followed by a much slower decay at larger distances from the center of the OD.

S3 Potential experimental realization

S3.1 DNA nanotechnology

The two most successful approaches to produce 3-dimensional periodic crystals made from DNA origami have been to use DNA origami polyhedra with single-stranded "sticky ends" at their vertices[11,12] or multi-arm DNA origami particles.[13] In the former approach each edge of the polyhedron is a multi-helix bundle and the single strands that mediate the interactions have a linker section and a complementary binding region. Due to the flexible linkers, the strands mediate little further angular constraints on the vertex-vertex interactions. As an example of the latter approach, in Ref. 13 4-arm DNA origami particles were able to assemble into a diamond lattice. Each arm consisted of a 24-helix bundle. At the centre of the design each arm splits into three 8-helix bundles that merge with the neighbouring arms.

"Insertions" and "deletions" were incorporated into the arms at these points to facilitate the required bending.[14] The symmetry of the design ensures the tetrahedrality of the particles. Six of the helices at the ends of the arms had short single-stranded extensions that mediated the interarm bonding; the pattern of extensions was designed to provide torsional control, in particular to ensure the "staggered" bonding geometry required for the diamond lattice.

Probably the most natural way to generate an equivalent of the patchy particles in the current paper that has the correct patch geometry is to use a single DNA origami particle with approximate (DNA origami have C_1 point group symmetry when all microscopic details are considered) icosahedral symmetry. In particular, the "wireframe" origami approach has been used to generate single DNA origami icosahedra with two helices along edge[15] and more recently with each edge being a 4-helix bundle.[12,16] The extra rigidity of the 4-helix bundle icosahedron probably makes it more suitable for higher-order assembly and it was one of the polyhedra used in Ref. 12 to generate DNA origami crystals.

The appropriate 7-edges of such a DNA icosahedron could be functionalized with single-stranded sticky ends to generate interactions that mimic the patchy particles considered here. It would make sense to use multiple sticky ends per edge. These would not all need to be at the centre of the edge, but could also be symmetrically positioned about the centre, as long as the sticky ends were specific enough to only interact with strands that are placed at the same distance from the centre. In this case, this would naturally generate a preference for edge-to-edge bonding, i.e. there would be some torsional-like constraint, the degree of which would depend on length of linkers. Note that we have shown that the patchy particles can assemble into an icosahedral QC both with and without a torsional component to the interactions and the edge-to-edge character of the interactions would give a preferred torsional angle that matches that required (Table S2). In contrast to this torsional constraint, the rocking motion about an axis parallel to the edges would be relatively free. Note also that this design could allow AB inter-patch interactions as well as AA and BB interactions, whilst also allowing the relative strengths of the BB and AA interactions to be fine tuned (for

example, by having different numbers of sticky ends on the A and B edges, or by varying the length of the complementary sections). Another potential hurdle is the self-complementarity of the interactions that are necessary in a one-component system, as the flexible linkers mean that intra-origami sticky-end binding might be more favourable than inter-origami binding. However, an approach to overcome this issue has been recently demonstrated that allows the assembly of crystals in one-component systems of DNA octahedra.[17]

An alternative approach might be to use 7-arm origami particles similar to the 4-arm particles in Ref. 13. However, it would be more difficult to get the correct geometry than for particles explicitly based on an icosahedral geometry. In particular, the inter-arm angles would need to be tuned via "insertions" and "deletions"[14] in order to best match the desired geometry. Modelling tools, such as oxDNA,[18,19] could play a significant role in this fine-tuning of the design. Postive features are that these particles are likely to be more rigid and have interactions that have a directionality that is more similar to our patchy particles. The interactions would also be torsionally-specific.

The above discussion is focussed on the 7P system. Although DNA origami analogues of the 10P particles could also be produced in a similar manner to suggested above, the requirement that the excluded volume limits the number of interactions with the B patches could be more problematic to implement. In particular, the icosahedra might not be sufficiently rigid to prevent this and the multi-arm particles would have a excluded volume that is very different from the spherical particles we consider here.

S3.2 Protein design

Another possibility to realize the quasicrystals in this paper would be through the tools of protein design, which have advanced considerably in recent years, especially as a result of approaches that leverage the power of machine learning.[20]

Although the propensity of many proteins to crystallize under the appropriate conditions has been vital to the field of protein structure determination, this is usually more an

unintended consequence of their well-defined structure. However, although comparatively rare, there are a significant number of examples of proteins that have evolved to crystallize for some functional purpose.[21,22] Similarly, proteins can be designed to form two- and three-dimensional periodic crystals.[23,24]

A recent example of the state-of-the-art in the design of proteins that can crystallize comes from Ref.[25] The approach is to first design proteins that can form high symmetry complexes that match the local symmetry of high symmetry sites in a crystal. Then intercomplex interactions are designed that allow these complexes to further assemble into the desired crystal. One example of this hierarchical approach was the creation of a diamond-like lattice in which a binary 24-protein complex with tetrahedral symmetry further assembles via intercomplex interactions along the four 3-fold axes of the tetrahedral complex.[25]

Like with DNA origami, one potential way to generate a particle with the correct "patch" geometry might be to start from a complex with icosahedral symmetry, there being a number of such designed examples[26–28] (as well as of course natural examples, for example, many virus capsids). The simplest ($T = 1$) involve 60 proteins. However, having intercomplex interactions associated with only a subset of the C_2 sites in the icosahedral complex would be a more complex task. One would need to first introduce some kind of programmed symmetry breaking where the icosahedron would be made of multiple homologous proteins with recoded interactions to generate the necessary specificity. Analogues of the C_{5v} P10 particle (rather than the C_s P7 particles) would likely be easier to achieve as it would require fewer distinct proteins due to the higher symmetry. Such programmed symmetry breaking has recently been used to generate a designed $T = 4$ icosahedra (and other polyhedra).[27] Furthermore, there has also been recent progress in generating designed multi-component complexes by the use of standardized protein building blocks.[28]

These challenges would seem to suggest that the realization of a protein icosahedral quasicrystal equivalent to those introduced in the current paper would be a harder task than one made from DNA origami, but one should not underestimate the rapid progress being

made in the field of protein design.

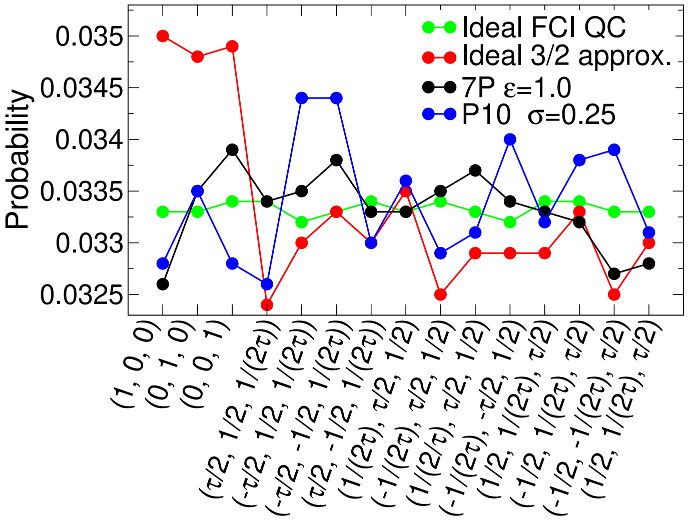

Figure S12: Integral of the bright spots in the BOODs along each of the 15 2-fold axes. Unsurprisingly, the BOOD for the annealed ideal FCI QC has the most uniform distribution. For the approximant there is a clear higher probability of bonds being oriented along the x, y and z axes that breaks the I_h symmetry.

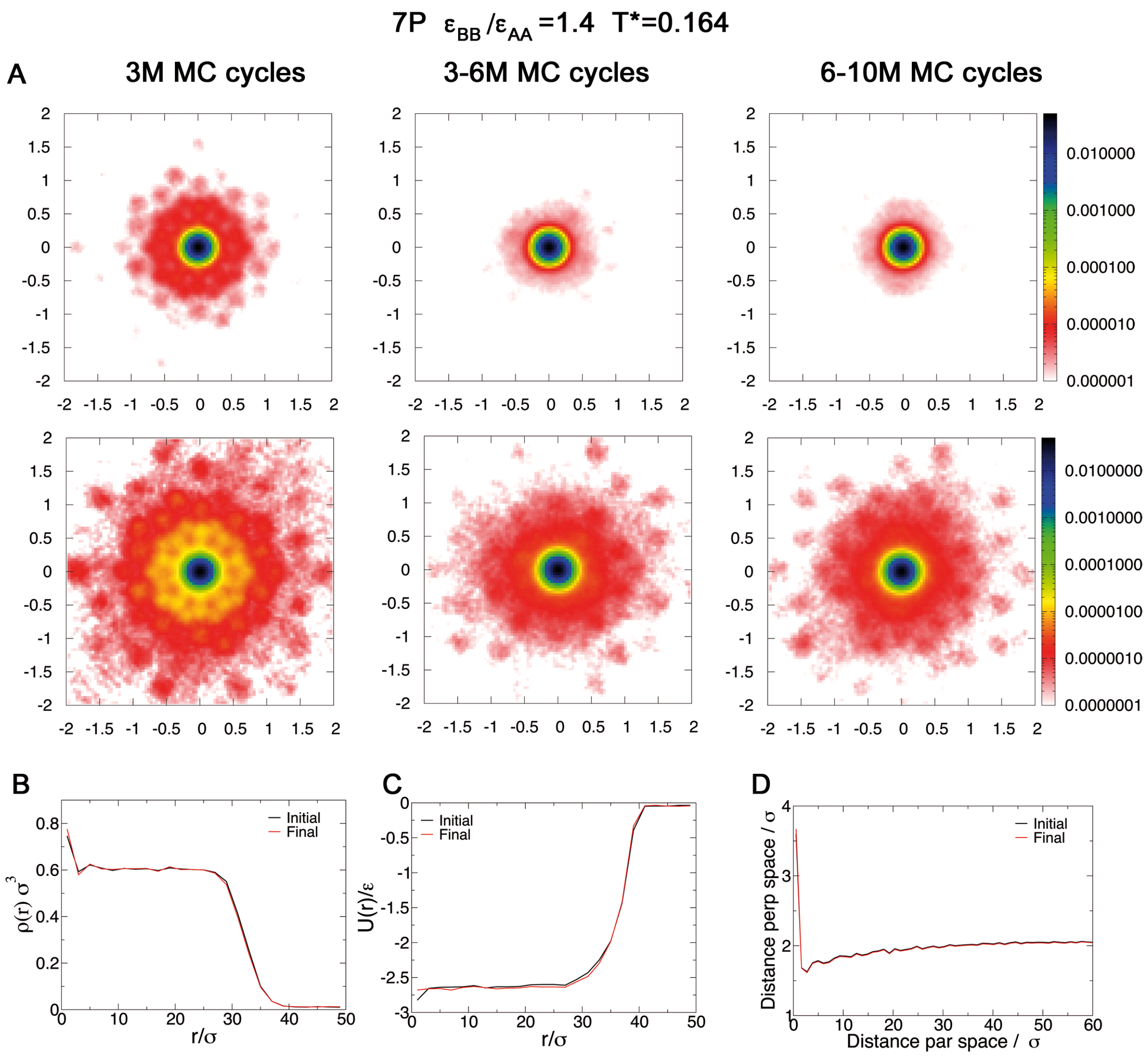

Figure S13: (A) Evolution of the Van Hove correlation function along a 10 million MC cycles trajectory of the 7P FCI model with $\epsilon_{BB}/\epsilon_{AA} = 1.4$ at T^*=0.164. The Van Hove correlation function was evaluated after 1 million MC cycles, averaging over 3–4 million MC cycles. Only particles within a radial distance of 27 σ of the centre of the condensed cluster were included in the evaluation of the Van Hove function to avoid surface effects. The two rows show the same data, but in the bottom row probabilities from 10^{-7} are shown (as compared to 10^{-6} in the upper row) to reveal more features of the diagrams. Particle mobility is significantly larger in the initial stage of the simulation (3 M MC cycles), after which it reaches a steady state. (B) The radial density of the cluster is virtually the same at the beginning and at the end of the 10 million MC cycles simulation. (C) The radial energy at the beginning and at the end is also very similar, with a very mild decrease at radii above 20 σ, close to the cluster surface. (D) The phason strain remains also almost invariant along the simulation.

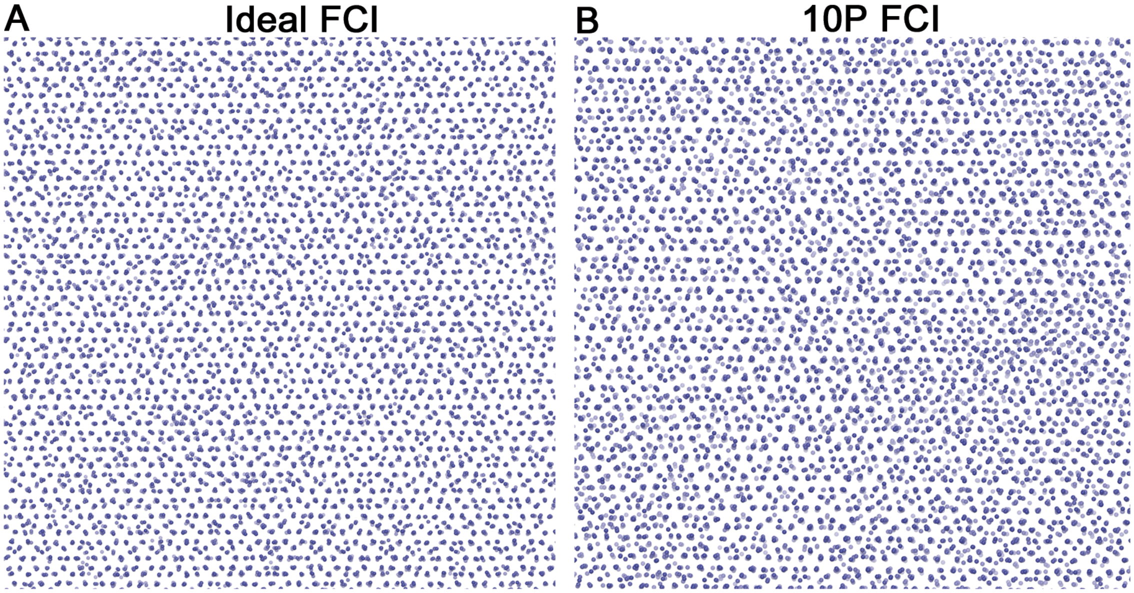

Figure S14: Comparison of the structures of the (A) tempered ideal FCI QC and (B) assembled 10P FCI QC. The 10P FCI QC possesses significantly greater phason disorder. This can be observed by inspecting the “lines” (of particles or gaps between particles) that run through the structures at 0, ±36°, ±72°, ±108° and ±154° to the horizontal (these are most easily seen viewed from a glancing angle). In the ideal QC each of these lines mostly run completely through the structure, but in the assembled they are often interrupted part way through by a “shift” in the structure (this shift is often termed a jag[10]). However, that the lines in a given direction are almost perfectly parallel provides evidence of the absence of dislocations (further confirmed by the lifting analysis).

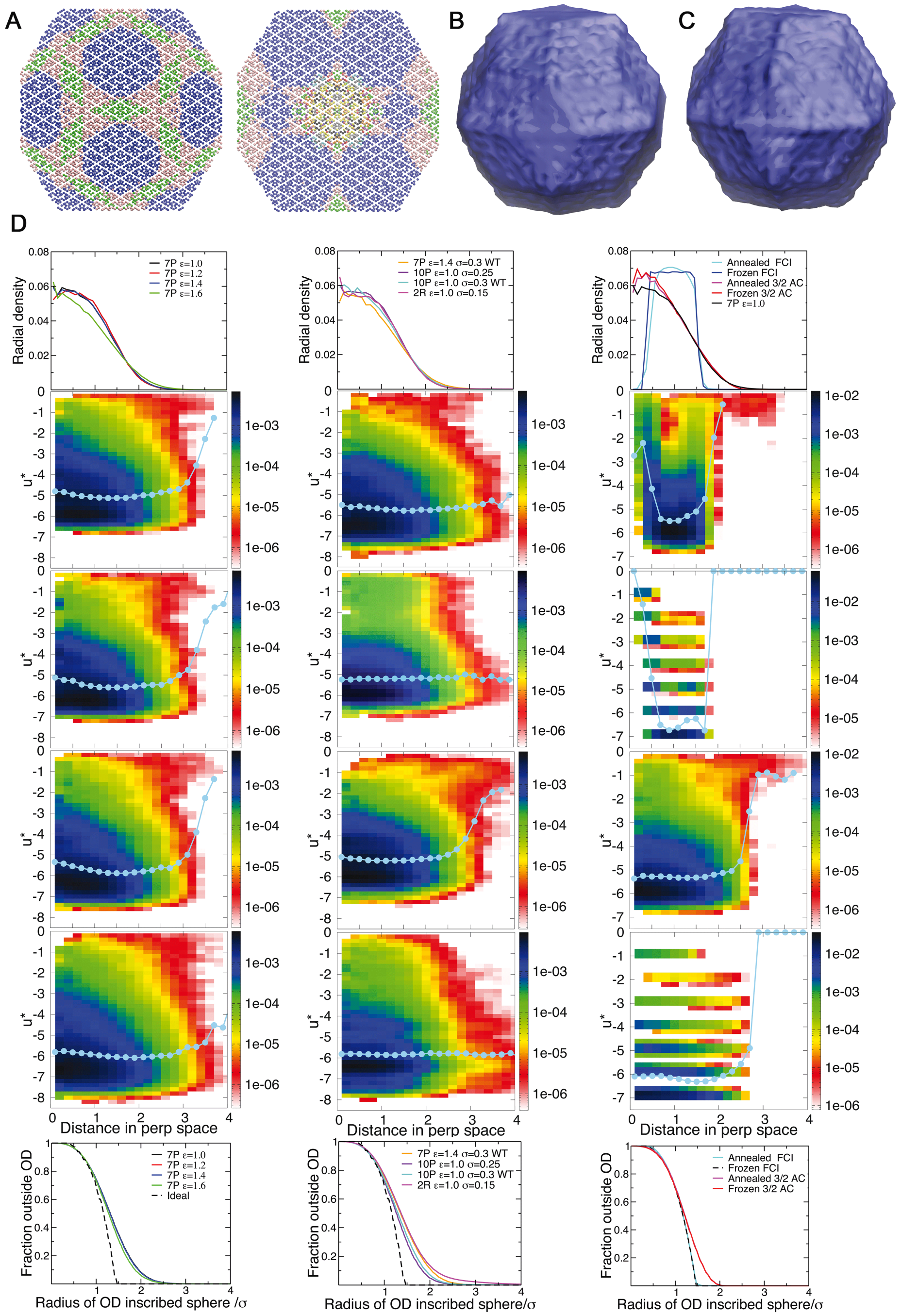

Figure S15: (A) Occupation domain of the ideal FCI QC. Each point is colored according to its local environment in real space (using the same color scheme as that in Fig. 1 of the main text). In the right-hand image the occupation domain is bisected to aid visualization. Isosurfaces of the occupation domain (B) in the ideal FCI QC and (C) in a tempered simulation starting from the ideal FCI QC. (D) Density of points in perpendicular space as a function of distance in perp space (top panel) and as a function of distance in perp space and particle energy (u^*). The energy-distance plots are shown from top to bottom in the same order as the legend of the radial density plots (top panel). Bottom panels show the fraction of points outside a 'scaled' occupation domain as a function of the radius of the inscribed sphere of the scaled dodecahedral occupation domain.

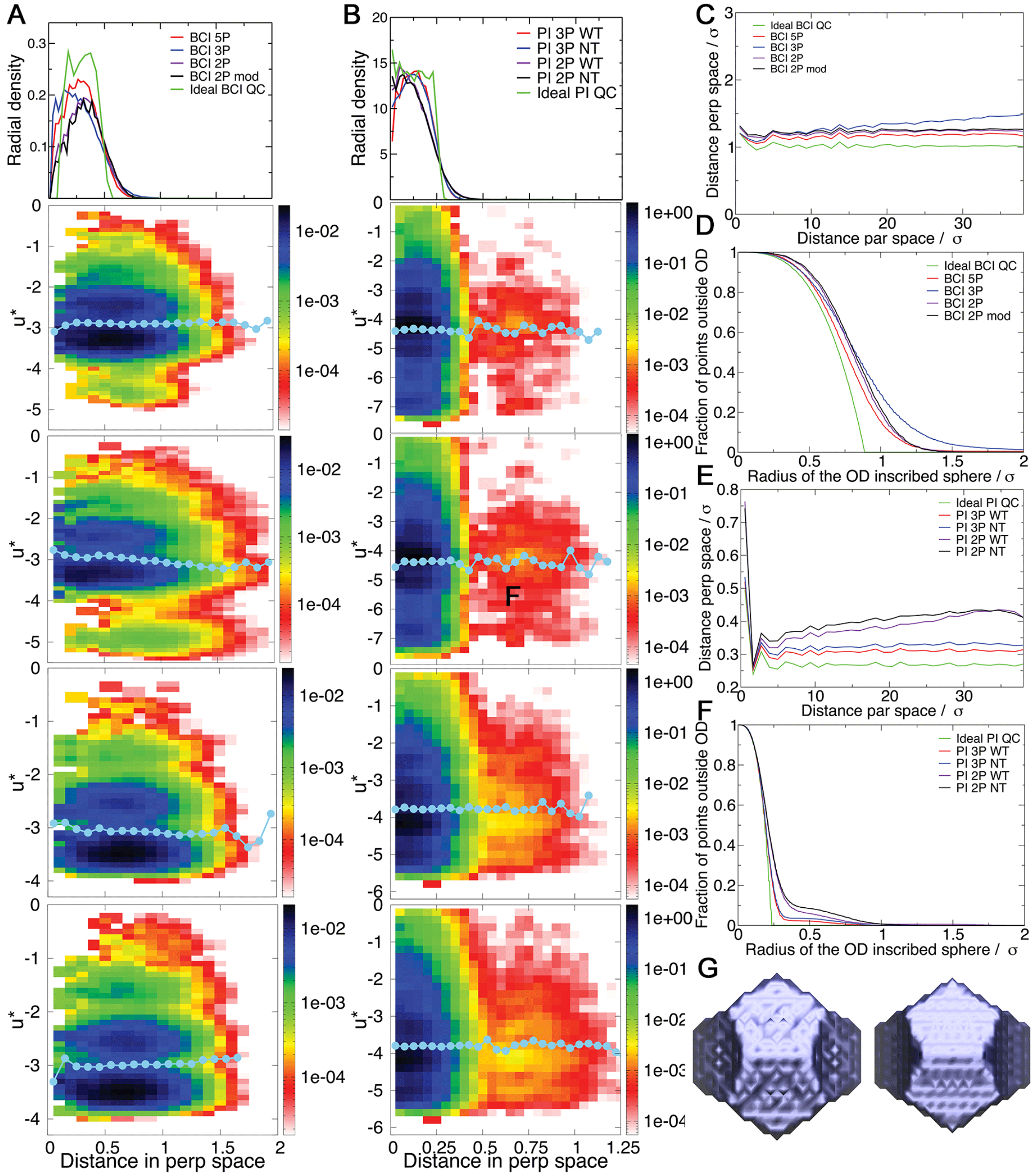

Figure S16: Lifting results for the BCI and PI QC reported in our previous work.[2] (A) and (B): Density of points in perpendicular space as a function of distance in perp space (top panel), and as a function of distance in perp space and particle energy (u^*). The energy-distance plots are shown from top to bottom in the same order as the legend of the radial density plots (top panel). (C) Phason strain for the BCI QC. (D) Fraction of points outside a 'scaled' occupation domain as a function of the radius of the inscribed sphere of the scaled dodecahedral occupation domain for the BCI QC model systems. This quantity was calculated by scaling the dodecahedral occupation domain by a given factor and measuring the number of atomic positions that fall outside this occupation domain. (E) Phason strain for the PI QC systems. (F) Fraction of points outside a 'scaled' occupation domain as a function of the radius of the inscribed sphere of the scaled dodecahedral occupation domain for the PI QC systems. (G) Shape of the occupation domain for the ideal BCI (left) and PI (right) QC. In both cases it has dodecahedral shape. Figures do not respect the relative size between both OD.

References

(1) Wilber, A. W.; Doye, J. P. K.; Louis, A. A.; Noya, E. G.; Miller, M. A.; Wong, P. Reversible self-assembly of patchy particles into monodisperse icosahedral clusters. *J. Chem. Phys.* **2007**, *127*, 085106.

(2) Noya, E. G.; Wong, C. K.; Llombart, P.; Doye, J. P. K. How to design an icosahedral quasicrystal through directional bonding. *Nature* **2022**, *596*, 367–371.

(3) Lifshitz, R. The square Fibonacci tiling. *J. Alloys Compd.* **2002**, *342*, 186–190.

(4) van der Linden, M. N.; Doye, J. P. K.; Louis, A. A. Formation of dodecagonal quasicrystals in two-dimensional systems of patchy particles. *J. Chem. Phys.* **2012**, *136*, 054904.

(5) Reinhardt, A.; Schreck, J. S.; Romano, F.; Doye, J. P. K. Self-assembly of two-dimensional binary quasicrystals: A possible route to a DNA quasicrystal. *J. Phys.: Condens. Matter* **2017**, *29*, 014006.

(6) Kowaguchi, A.; Mehta, S.; Doye, J. P. K.; Noya, E. G. A patchy-particle 3-dimensional octagonal quasicrystal. arXiv:2408.05003.

(7) Wilber, A. W.; Doye, J. P. K.; Louis, A. A. Self-assembly of monodisperse clusters: Dependence on target geometry. *J. Chem. Phys.* **2009**, *131*, 175101.

(8) Ouldridge, T. E.; Louis, A. A.; Doye, J. P. K. Extracting bulk properties of self-assembling systems from small simulations. *J. Phys.: Condens. Matter* **2010**, *22*, 104102.

(9) Keys, A. S.; Glotzer, S. C. How do quasicrystals Grow? *Phys. Rev. Lett.* **2007**, *99*, 235503.

(10) Socolar, J. E. S. Phason strain in quasicrystals. *J. Phys. Colloques* **1986**, *47*, C3–217–C3–226.

(11) Tian, Y.; Lhermitte, J. R.; Bai, L.; Vo, T.; Xin, H. L.; Li, H.; Li, R.; Fukuto, M.; Yager, K. G.; Kahn, J. S.; Xiong, Y.; Minevich, B.; Kumar, S. K.; Gang, O. Ordered three-dimensional nanomaterials using DNA-prescribed and valence-controlled material voxels. *Nat. Mater.* **2020**, *19*, 789–796.

(12) Liu, H.; Matthies, M.; Russo, J.; Rovigatti, L.; Narayanan, R. P.; Diep, T.; McKeen, D.; Gang, O.; Stephanopoulos, N.; Sciortino, F.; Yan, H.; Romano, R.; Šulc, P. Inverse design of a pyrochlore lattice of DNA origami through model driven experiments. *Science* **2024**, *384*, 776–781.

(13) Posnjak, G.; Yin, X.; Butler, P.; Bienek, O.; Dass, M.; Sharp, I. D.; Liedl, T. Diamond photonic crystals assembled from DNA origami. *Science* **2024**, *384*, 781–785.

(14) Dietz, H.; Douglas, S. M.; Shih, W. M. Folding DNA into twisted and curved nanoscale shapes. *Science* **2009**, *325*, 725–730.

(15) Veneziano, R.; Ratanalert, S.; Zhang, K.; Zhang, F.; Yan, H.; Chiu, W.; Bathe, M. Designer nanoscale DNA assemblies programmed from the top down. *Science* **2016**, *352*, 1534.

(16) Zhang, J.; Xu, Y.; Huang, Y.; Sun, M.; Liu, S.; Wan, S.; Chen, H.; Yang, C.; Yang, Y.; Song, Y. Spatially patterned neutralizing icosahedral DNA nanocage for efficient SARS-CoV-2 blocking. *J. Am. Chem. Soc.* **2022**, *144*, 13146–13153.

(17) Zhou, Z.; Ji, M.; Yu, Y.; Wang, L.; Dai, L.; Yan, X.; Xie, X.; Ma, N.; Huang, S.; Tian, Y. Phase behavior modulation of a unary DNA origami system through allosteric stimuli. *Nano Lett.* **2024**, *24*, 12263–12270.

(18) Snodin, B. E. K.; Randisi, F.; Mosayebi, M.; Šulc, P.; Schreck, J. S.; Romano, F.; Ouldridge, T. E.; Tsukanov, R.; Nir, E.; Louis, A. A.; Doye, J. P. K. Introducing improved structural properties and salt dependence into a coarse-grained model of DNA. *J. Chem. Phys.* **2015**, *142*, 234901.

(19) Snodin, B. E. K.; Schreck, J. S.; Romano, F.; Louis, A. A.; Doye, J. P. K. Coarse-grained modelling of the structural properties of DNA origami. *Nucleic Acids Res.* **2019**, *47*, 1585–1597.

(20) Winnifrith, A.; Outeiral, C.; Hie, B. L. Generative artificial intelligence for *de novo* protein design. *Curr. Opin. Struct. Biol.* **2024**, *86*, 102794.

(21) Doye, J. P. K.; Poon, W. C. K. Protein crystallization *in vivo*. *Curr. Opin. Colloid In.* **2006**, *11*, 40–46.

(22) Schönherr, R.; Rudolph, J. M.; Redecke, L. Protein crystallization in living cells. *Biol. Chem.* **2018**, *399*, 751–772.

(23) Yeates, T. O. Geometric principles for designing highly symmetric protein nanomaterials. *Annu. Rev. Biophys.* **2017**, *46*, 23–42.

(24) Zhu, J.; Avakyan, N.; Kakkis, A.; Hoffnagle, A. M.; Han, K.; Li, Y.; Zhang, Z.; Choi, T. S.; Na, Y.; Yu, C.-J.; Tezcan, F. A. Protein assembly by design. *Chem. Rev.* **2021**, *121*, 13701–13796.

(25) Li, Z. et al. Accurate computational design of three-dimensional protein crystals. *Nat. Mater.* **2023**, *22*, 1556–1563.

(26) Lutz, I. D.; Wang, S.; Norn, C.; Borst, A. J.; Zhao, Y. T.; Dosey, A.; Cao, L.; Li, Z.; Baek, M.; King, N. P.; Ruohola-Baker, H.; Baker, D. Top-down design of protein architectures with reinforcement learning. *Science* **2023**, *380*, 266–273.

(27) Lee, S.; Kibler, R. D.; Hsia, Y.; Borst, A. J.; Philomin, A.; Kennedy, M. A.; Stoddard, B.; Baker, D. Four-component protein nanocages designed by programmed symmetry breaking. *Nature* **2025**, *638*, 546–552.

(28) Huddy, T. F. et al. Blueprinting extendable nanomaterials with standardized protein blocks. *Nature* **2024**, *627*, 898–904.
